# Attention and executive delays in early childhood: a meta-analysis of neurodevelopmental conditions

**DOI:** 10.1038/s41380-024-02802-3

**Published:** 2024-11-03

**Authors:** Dabin Lee, Kelsie A. Boulton, Carter Sun, Natalie L. Phillips, Martha Munro, Fiona Kumfor, Eleni A. Demetriou, Adam J. Guastella

**Affiliations:** 1 Clinic for Autism and Neurodevelopmental (CAN) research, Brain and Mind Centre, Children’s Hospital Westmead Clinical School, Faculty of Medicine and Health, University of Sydney, Camperdown, Australia; 2 Child Neurodevelopment and Mental Health Team, Brain and Mind Centre, University of Sydney, Camperdown, Australia; 3 School of Psychology and Brain and Mind Centre, University of Sydney, Camperdown, Australia

**Keywords:** Autism spectrum disorders, Prognostic markers, ADHD

## Abstract

The objective of this review was to evaluate attention and executive function performance in children with neurodevelopmental conditions across the first 5 years of life, compared to neurotypical peers. MEDLINE, EMBASE, and PsycINFO databases were searched until June 30, 2023, and studies comparing attention or executive function between children with (or at risk for) neurodevelopmental conditions and neurotypical (or low risk) peers, 0 to 5 years old, were included. Of the 4338 studies identified, 111 studies with 12292 participants were included in the meta-analysis. The qualitative analysis of brain development included 5 studies. Primary outcomes were the standardised mean difference (Hedges’ g) in attention and executive function between groups. Meta-regressions examined moderating effects of age, biological sex, diagnosis, and measure type. Children with neurodevelopmental conditions showed small delays in attention (*n* = 49 studies, *k* = 251 outcomes, *g* = 0.36, 95% CI 0.23-0.48, *p* < 0.001) and moderate delays in executive function (*n* = 64 studies, *k* = 368 outcomes, *g* = 0.64,95% CI 0.53–0.76, *p* < 0.001). Attention and executive function delays could not be identified in the first year (equivalence tests, *p* < 0.001), small to moderate delays were found in toddlerhood and moderate delays by preschool. Delays identified were largely transdiagnostic, although there was some evidence of diagnosis-specific delays for attention and moderation by measure type (informant rating vs performance-based vs physiological). Qualitative analysis described how delays were underpinned by a divergence of brain development in medial prefrontal regions. These findings highlight the potential of using attention and executive measures to detect delay and to intervene in neurodevelopmental conditions early in life.

## Introduction

In the first years of life, children develop core cognitive capacities that are crucial for lifelong skills. Attention and executive function (EF) are believed to be particularly important, as they underpin adaptive and goal-directed behaviour [1]. These capacities are associated with emotional and behavioural functioning, social competence, academic achievement, and vocational opportunities over the lifespan [2–6].

Delays in attention and EF have been associated with various neurodevelopmental conditions (NDCs) and atypical brain development [7, 8]. Evidence of impairments have been shown across autism, attention-deficit/hyperactivity disorder (ADHD), intellectual developmental disorders (IDD), specific learning disorders (SLD) and communication disorders [9–13]. This has led to more recent work showing that attention and EF delays are transdiagnostic features of NDCs [14, 15]. However, it remains unclear whether these delays can be identified in early childhood. Given the established support for a transdiagnostic approach to neurodevelopment [14–17], this review will collate evidence across the spectrum of NDCs to examine whether attention and EF delays can be identified in the first years of life. Such knowledge may pave the way for very early detection and intervention opportunities even prior to a diagnosis [18].

## Typical development of early executive function

In typical development [19], EF has been proposed to emerge in the first years of life, with core, or simple, EF components preceding the development of complex EF components (see Table 1). Working memory, response inhibition and shifting are widely considered core EF components [1, 19]. These components emerge in their simplest form from very early in development, are built upon across the first years of life [20], and maintain a stable developmental trajectory throughout the school years [21]. In parallel to this development of core EF components, attention is believed to form the developmental groundwork for EF [19], with studies showing that attention performance in the first 6 months of life may be predictive of later EF [22, 23]. Indeed, in early infancy, attention is typically the primary focus of assessment as other EF capacities have not yet developed [19, 23]. As such, the assessment of EF in childhood, particularly in early infancy, should include consideration of attention as a construct and an ability that may underpin later EF development. Both attention and EF development are underpinned by maturation of the prefrontal cortex (PFC) and closely related regions [24–26].

**Table 1:** Executive Function Domains and their Proposed Early Developmental Trajectory in Typical Development [19].

EF domain	Description	Early Developmental trajectory	Example EF task and outcome
Simple working memory	Holding information in mind and over delay	Emerges in the first 6 months of life, followed by steady development	**Delayed response task:** Child is given an object which is withdrawn and hidden in one of two (or more) locations, and child must find object after a delay.
Complex working memory	Holding in mind and updating/manipulating information	Builds upon simple working memory and emerges in the second year of life, followed by steady development	**Invisible displacement**: An object is hidden under a small container. The container is moved under one of two larger containers, and the object is left inside. Child is then shown the empty small container. After a delay, the child searches for the object.
Simple response inhibition	Withholding/delay of prepotent response	Emerges in the first year of life, followed by steady development	**Go/No-go task:** Child is required to respond by pressing a button when a ‘go’ signal appears and inhibits responding when a ‘no-go’ signal appears. The ability to inhibit responses on ‘no-go’ trials is the behaviour measured.
Complex response inhibition	Holding a rule in mind, responding according to this rule, and inhibiting a prepotent response	Builds upon simple response inhibition and emerges in the second year of life, followed by steady development	**Reverse categorization**: Child is required to sort big blocks into little bucket and little blocks into big bucket.
Response shifting	Forming an arbitrary S-R set in the first phase and shifting to a new S-R set in the second phase	Emerges in the second year of life, followed by steady development	**A-not-B task:** In sight of child, an object is hidden at Location A, and child must find the object after a delay. Once child successfully finds the object at Location A in a number of consecutive trials, the object is hidden at location B. Child must find the object at Location B.
Attention shifting	Similar to response shifting except the first mental set involves attention to one aspect of the stimuli and the shifting phase involves shifting attention to a new aspect of the stimuli.	Emerges in the second year of life, followed by steady development	**Dimension Card Change Sort:** Child is shown cards depicting stimuli that can be sorted according to colour or shape. Child must sort according to one dimension (e.g., colour) and then shift to sort according to the other dimension (e.g., shape).
Higher order EF	Planning, reasoning, and problem-solving skills, which build on working memory, response inhibition and set shifting	Builds up on working memory, response inhibition and set-shifting and emerges after the third year of life, followed by steady development	**Tower of London task:** Child is presented with coloured disks stacked vertically in three possible positions. Child must move the disks one at a time until they match a given configuration.

*EF* executive function, *S-R* stimulus-response.

## Objective

This review will take a transdiagnostic approach to determine whether and when children with (or at risk of) NDCs demonstrate delays in attention and EF in the first 5 years of life, compared to neurotypical (or low-risk) peers. To do this, we have adopted a theoretical framework of early EF development [19]. Given the critical role of attention as a developmental precursor of EF, we evaluated the evidence for overall attention difficulties in children with NDCs. We then took a two-pronged approach to studying EF. First, we examined the overall EF impairment in children with NDCs. Second, we explored how the delays may manifest across the simple and complex EF components (see Table 1). A second objective was to examine the different factors (age group, sex distribution, NDC diagnosis, type of measure) that may moderate attention and EF delays. Finally, we aimed to explore whether the early attention and EF impairments in children with NDCs are related to divergences in their structural and/or functional brain development.

## Methods

This review was conducted in accordance with the Meta-analyses Of Observational Studies in Epidemiology (MOOSE) guidelines and was registered with PROSPERO (CRD42022333295).

### Search strategy

Databases including MEDLINE, EMBASE, and PsycINFO were searched on June 30, 2023, using a combination of keywords and Medical Subjects Headings (MeSH; exploded to retrieve citations associated with a wide range of more narrow terms) relating to NDCs (e.g., autism) AND attention/EF domains (e.g., working memory; Supplementary Table 1). Additional articles were sought by reviewing references and contacting authors for unpublished data. Articles were imported and managed using Endnote and Covidence systematic review software (Veritas Health Innovation, Melbourne, Australia).

### Eligibility criteria

Original peer-reviewed research studies (including cross-sectional, cohort, longitudinal, randomised, and non-randomised controlled trials) published in the English language were included. Only baseline data were extracted from longitudinal and controlled trials. Studies were included if participants were (i) aged 0 to 5 years old and (ii) diagnosed with an NDC using a valid assessment tool (e.g., autism diagnostic observation scale, autism diagnostic interview-revised, diagnostic interview schedule for children – young child) and/or following clinical evaluation or identified to be at risk for an NDC. Criteria typically used to classify young children as at risk for an NDC include familial history (e.g., older sibling with autism or ADHD) or exposure to identified risk factors (e.g., prenatal exposure to alcohol). Included studies had at least one physiological, performance-based, informant report or brain-based measure of non-social attention or EF. Studies without neurotypical or low-risk controls, or normative data were excluded. Detailed eligibility criteria are provided in Supplementary Methods 1.

### Selection and data extraction process

The search strategy identified a total of 4338 potentially eligible studies (Supplementary Fig. 1). Two independent reviewers (K.A.B., N.P.) conducted an initial screening of the title and abstract using Covidence, resulting in 650 articles for full-text review. Full-text reviews were conducted by two independent reviewers (D.L., E.A.D.), with the second reviewer performing 20% of the reviews. This resulted in 116 articles for data extraction, which included 1 unpublished dataset. Data, including outcome measures and participant demographics, were extracted by one reviewer (D.L.; Supplementary Methods 2), and 20% of the data were cross-checked by a second independent reviewer (M.M.). The inter-rater agreement rate was 99.6%, indicating substantial agreement. All reviewers hold a minimum qualification of a post-graduate degree. When studies included more than one attention or EF outcome, all relevant outcomes were extracted to minimise selective data extraction. Disagreements were resolved by making a consensus decision or through consultation with a third independent reviewer.

### Study variables

The overall EF analysis included all EF outcomes from performance-based measures and the composite scores from informant ratings; there were no EF outcomes evaluated using physiological measures. For the analysis of separate EF components, EF outcomes were coded according to two different coding schemes depending on the type of measure, based on the postulation that performance-based measures and informant ratings capture two distinct aspects of EF [27]. EF outcomes from performance-based measures were coded into 7 separate EF domains [19] (see Table 1). EF outcomes from informant ratings were coded based on the established subscales and factors of the questionnaires. As attention was included as an EF precursor, attention outcomes were not coded into separate domains. Four variables were selected for moderator analyses: age group, sex distribution, NDC diagnosis and type of measure. Three age groups were selected for analysis: infancy (0–1 year old), toddlerhood (1 – 3 years old), and the preschool period (3 – 5 years old), as early EF development appears to be demarcated by these three developmental phases [19, 28, 29]. Detailed descriptions of study variables are provided in Supplementary Methods 3.

### Data Analysis

#### Quantitative Analysis

Quantitative analyses of the extracted measures were conducted using the “metafor” (Version 4.2-0) and “meta” (Version 6.5-0) packages in RStudio (2023.06.1 Build 524) and R (version 4.2.1) and the “rma.mv” function for between-study multivariate random-effects model, as recommended by the Cochrane Handbook for Systematic Reviews of Interventions [30]. Effect sizes were calculated as standardised mean differences (Hedges’ *g*) of the outcome measure between the NDC and neurotypical groups using the “escalc” function. A positive effect size indicated that the neurotypical group performed better than the NDC group. Data analyses for attention and overall EF were planned a priori. First, a meta-analysis of overall attention was conducted. Second, meta-analyses of EF were conducted on two levels: (1) overall EF; and (2) individual EF components. Two separate analyses were completed for individual EF components assessed using performance-based measures and informant ratings. The data analysis was only conducted if there were at least 3 studies per outcome measure, and studies that used the same cohort were nested to reduce potential biases. Given there are limitations to some methods for controlling for publication bias, we conducted three analyses. Small study bias and publication bias were assessed using Egger’s regression test [31] to evaluate funnel plot asymmetry. The Trim-and-fill method was also conducted to impute missing studies, and to estimate the adjusted effect size. In addition, a robust Bayesian meta-analysis was conducted using the “RoBMA” (Version 3.1.0) package. Multi-variate meta-regressions were conducted to explain the heterogeneity for attention and EF with the moderator variables (age, sex distribution, NDC diagnosis and type of measure). The magnitudes of Hedges’ *g* effect sizes were interpreted, where >0.20, between >0.50 and <0.80, and ≥0.80 are respectively described as small, moderate, and large. Between-study heterogeneity was assessed using the τ^2^ statistic. Two one-sided t-tests (TOST) were performed using the “TOSTmeta” function in the “TOSTER” package to assess the statistical and clinical equivalence between groups. The analysis utilised a predefined margin of equivalence, with a lower equivalence bound of -0.20 and an upper equivalence bound of 0.20, based on the smallest effect size of interest [32, 33].

#### Qualitative analysis

A qualitative synthesis was performed for the studies addressing the question of whether the early attention and EF impairments in children with NDCs occur alongside atypical brain development. A qualitative synthesis was selected because the high heterogeneity across the studies was expected to preclude an informative quantitative synthesis.

### Quality assessment

Study quality was assessed by two independent assessors using the Joanna Briggs Institute Critical Appraisal Tool, with the second assessor performing 20% of the quality assessment [34].

## Results

### Quantitative analysis

#### Meta-analysis

**Fig. 1:**
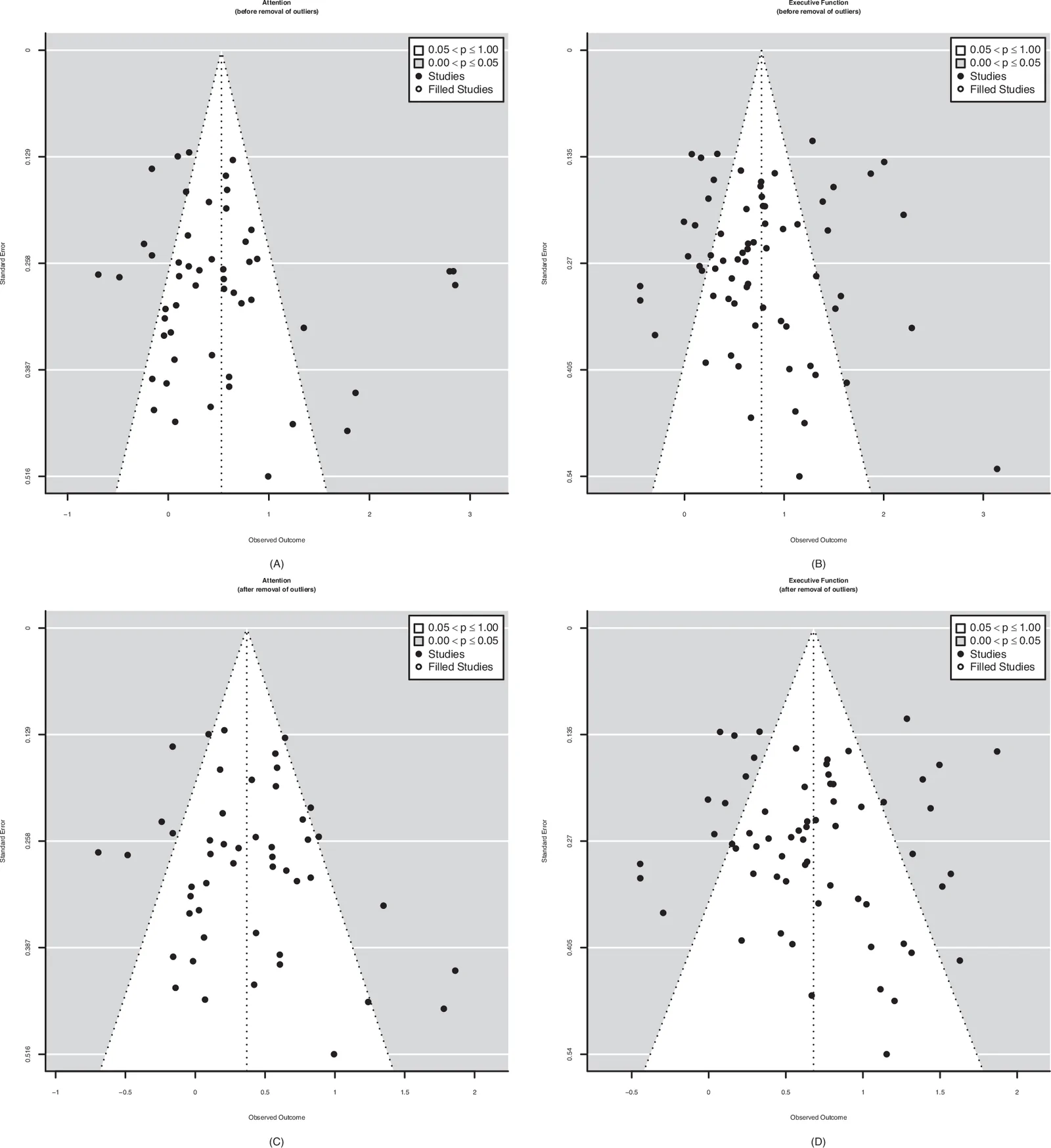
Funnel plots for the included studies. Funnel plots for asymmetry analysis using the Trim and Fill method. **A** Includes all eligible studies reporting attention measures. **B** Includes all studies reporting executive function measures. **C** Attention studies with outliers (Hedges’ *g* > 2) excluded. **D** Executive function studies with outliers (Hedges’ *g* > 2) excluded. The vertical dashed line represents the nested mean effect size for each study, and the outer dashed lines show the 95% confidence intervals. Grey shaded areas indicate statistically significant differences (*p* < 0.050), while the white area represents non-significant differences (*p* > 0.050). The *p* values are derived from two-sided random-effects models.

The main quantitative analysis included a total of 92 unique cohorts from 111 included studies (Supplementary Table 2), with 12292 participants (NDC: 4699, Control: 7593). The NDC group showed significant impairments in attention (*n* = 52, *k* = 260, *g* = 0.50, 95% CI 0.31–0.70, *p* < 0.001, *τ*^2^ = 0.47, prediction interval -0.86–1.86) and EF (*n* = 68, *k* = 397, *g* = 0.73, 95% CI 0.59–0.87, *p* < 0.001, *τ*^2^ = 0.31, prediction interval -0.36–1.82). The funnel plot and Egger’s regression test presented significant evidence of publication bias for both attention (*β* = 0.53, *p* < 0.001) and EF (*β* = 0.77, *p* < 0.001; Fig. 1A and B). A trim and fill analysis was conducted, and no missing studies were imputed for either attention or EF. There was a reduction in effect size following the removal of outliers (Hedges’ *g* > 2 [10], Supplementary Table 3) for both attention (*n* = 49, *k* = 251, *g* = 0.36, 95% CI 0.23–0.48, *p* < 0.001, *τ*^2^ = 0.17, prediction interval -0.45–1.17, Supplementary Fig. 2, Bayesian Model results in Supplementary Results 1) and EF (*n* = 64, *k* = 368, *g* = 0.64, 95% CI 0.53–0.76, *p* < 0.001, *τ*^2^ = 0.19, prediction interval -0.23–1.51, Supplementary Fig. 3, Bayesian Model results in Supplementary Results 1). Publication bias was reduced following the outlier removal for both attention (*β* = 0.37, *p* < 0.001) and EF (*β* = 0.68, *p* < 0.001; Fig. 1C and D). An additional analysis excluding studies with high-risk NDC groups produced consistent results for both attention (*n* = 42, *k* = 21, *g* = 0.58, 95% CI 0.36–0.83, *p* < 0.001, *τ*^2^ = 0.58, prediction interval -0.91–2.10) and EF (*n* = 61, *k* = 304, *g* = 0.82, 95% CI 0.67–0.97, *p* < 0.001, *τ*^2^ = 0.33, prediction interval -0.32–1.96).

**Fig. 2:**
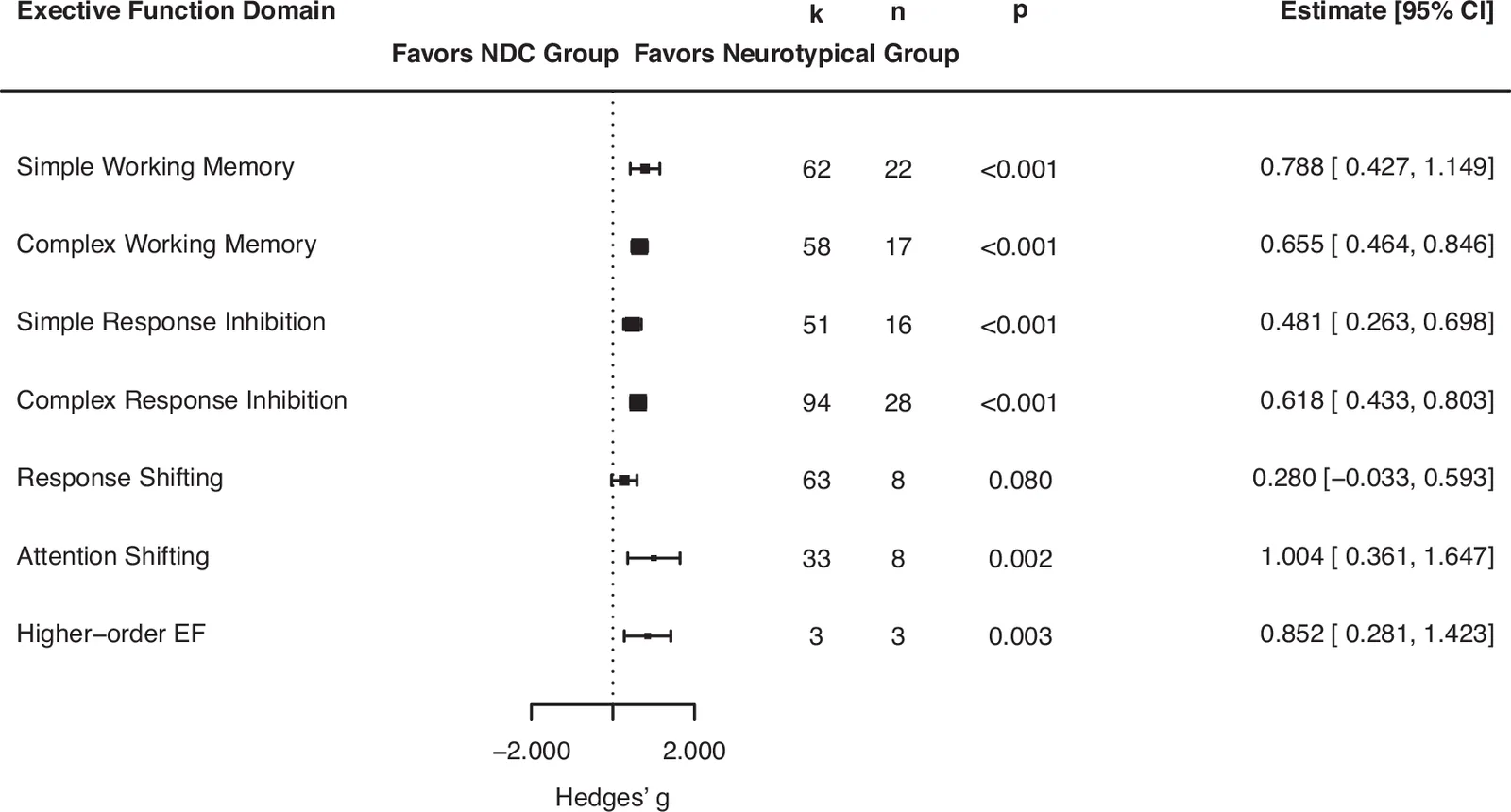
Differences in EF between children with NDCs and neurotypical peers across EF domains assessed using performance-based measures. k number of outcomes, n number of studies, NDC neurodevelopmental condition, CI confidence interval, EF executive function.

Separate analyses conducted for each EF domain from the performance-based measures revealed significant impairment in the NDC group across all domains except for response shifting (*n* = 8, *k* = 63, *p* = 0.080, see Fig. 2), with effect sizes ranging from moderate to large magnitudes. The analyses conducted for each EF component from informant ratings demonstrated significant impairment in the NDC group across all EF subscales and factors, all with large effect sizes (Supplementary Table 4).

#### Moderator analysis

Moderator analyses were conducted for attention and EF (Supplementary Tables 5 and 6).

##### Age groups

**Fig. 3:**
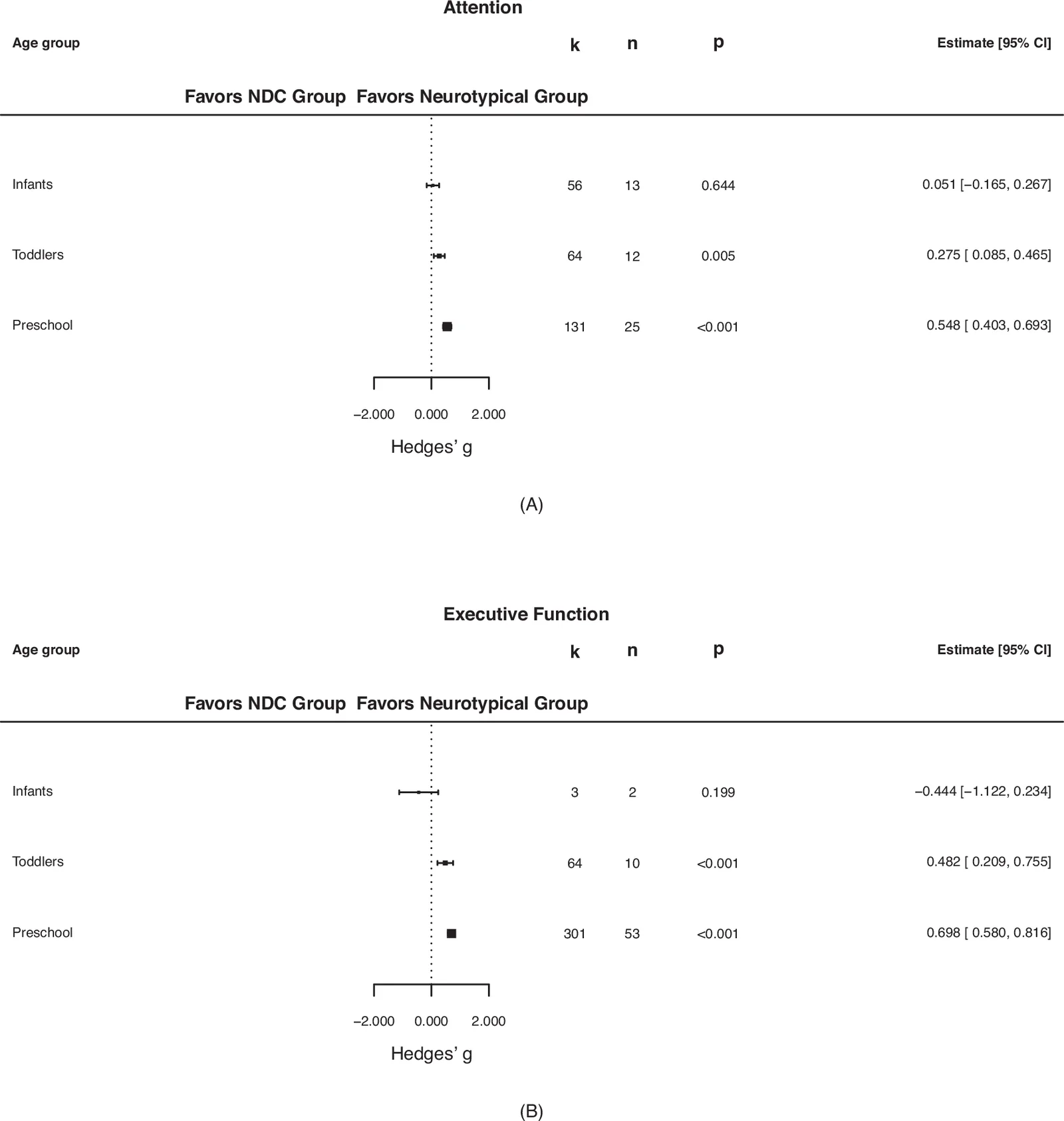
Difference in **A** Attention and **B** EF between children with NDCs and neurotypical peers across age groups. k number of outcomes, n number of studies, NDC neurodevelopmental condition, CI confidence interval, EF executive function.

The moderator analyses revealed a significant effect of age groups on both attention (*n* = 49, *k* = 251, *Q*_(df=3)_ = 16.67, *p* < 0.001) and EF (*n* = 64, *k* = 368, *Q*_(df=2)_ = 12.18, *p* = 0.002). This was driven by: (i) a lack of significant impairment in the NDC group in infancy, which emerged in toddlerhood and the preschool period; and (ii) the increase in the magnitude of delay from toddlerhood to the preschool period (see Fig. 3). Equivalence testing showed no significant impairment in the NDC group during infancy. The TOST results suggested no statistically significant difference from zero, but also did not establish statistical and clinical equivalence between the NDC and control groups in infancy for both attention (*n* = 13, *Z* = −1.40, 90% CI [−0.13, 0.23], *p* = 0.081, given equivalence bounds of −0.20 and 0.20) and EF (*n* = 2, *Z* = −0.71, 90% CI [−1.01, 0.13], *p* = 0.760, given equivalence bounds of −0.20 and 0.20). Additionally, the null hypothesis (there is a difference between groups) was statistically non-significant (attention: *Z* = 0.47, 95% CI [−0.16, 0.26], *p* = 0.640; EF: *Z* = −1.28, 95% CI [-1.12, 0.23], *p* = 0.199), indicating no difference between groups.

##### Sex distribution

There was no significant effect by sex for either attention (*n* = 46 studies, *k* = 239 outcomes, *Q*_(df=1)_ = 0.03, *β* = 0.0003, 95% CI −0.004–0.004, *p* = 0.864) or EF (*n* = 61 studies, *k* = 348 outcomes, *Q*_(df=1)_ = 0.74, *β* = 0.001, 95% CI −0.002–0.005, *p* = 0.389).

##### NDC diagnosis

Conditions represented in the final cohort of studies included: autism (attention: *n* = 17, EF: *n* = 19), communication disorders (attention: *n* = 7, EF: *n* = 15), ADHD (attention: *n* = 6, EF: *n* = 12), genetic disorders (attention: *n* = 8, EF: *n* = 9), high-risk autism (attention: *n* = 11, EF: *n* = 4), intellectual developmental disorder (attention: *n* = 3, EF: *n* = 1), cerebral palsy (attention: *n* = 2, EF: *n* = 2), specific learning disorder (attention: *n* = 2, EF: *n* = 2), mixed NDCs (attention: *n* = 1, EF: *n* = 3), motor disorders (EF: *n* = 1), high-risk ADHD (EF: *n* = 1), FASD (EF: *n* = 1), high-risk FASD (attention: *n* = 1, EF: *n* = 1), comorbid ADHD and oppositional defiant disorder (attention: *n* = 1, EF: *n* = 3), high-risk communication disorders (attention: *n* = 2, EF: *n* = 2). The diagnosis was found to be a significant moderator for both attention (*n* = 49; *k* = 251, *Q*_(df=10)_ = 34.60, *p* < 0.001) and EF (*n* = 64, *k* = 368, *Q*_(df=14)_ = 38.96, *p* < 0.001), which resulted from different significance results and effect sizes of delay across different NDC groups. Importantly, however, most of the NDC groups that did not demonstrate a significant delay in attention/EF were characterised by a small number of studies (*n* < 3). Given this, the moderator analyses were repeated after the removal of NDC groups with less than 3 studies (see Table 2). NDC diagnosis remained a significant moderator for attention only. For attention, moderate attentional delays were found for the communication disorder and ADHD groups, while the IDD, high-risk autism and the genetic conditions group showed small delays. The autism group (*p* = 0.088, see Table 2) did not show significant attentional delay. EF delays were evident across all NDC groups including communication disorders, autism, ADHD, genetic conditions, high-risk autism, mixed NDCs and comorbid ADHD and oppositional defiant disorder, all of which exhibited moderate to large effect sizes except for the high-risk autism group, where there was evidence of a small effect.

**Table 2:** Moderator effect of NDC Diagnosis after the removal of NDC groups with less than 3 studies.

Attention							
Test of moderator: NDC diagnosis				QM (df = 5) = 13.55, *p* = 0.02 τ^2^ = 0.17 (*n* = 44, *k* = 232)			
	IDD	Communication disorders	Autism	ADHD	Genetic conditions	High-risk autism	
***n*** **(k)**	3 (14)	7 (22)	17 (66)	6 (39)	8 (60)	11 (31)	
**Hedges’ g (CI 95%)**	0.309 (0.065–0.553)	0.748 (0.403–1.093)	0.159 (−0.023–0.342)	0.643 (0.297–0.989)	0.298 (0.047–0.549)	0.308 (0.099–0.517)	
***p*-value**	0.013	<0.001	0.088	<0.001	0.020	0.004	
**Executive Function**							
**Test of moderator: NDC diagnosis**				**QM (df = 6) = 7.75,** ***p*** **= 0.26 τ**^**2**^** = 0.20** **(** ***n*** **= 56,** ***k*** **= 300** **)**			
	**Mixed NDCs**	**Communication disorders**	**Autism**	**ADHD**	**Genetic conditions**	**High-risk autism**	**Comorbid ADHD and oppositional defiant disorder**
	3 (24)	15 (74)	19 (76)	12 (70)	9 (32)	4 (8)	3 (16)
**Hedges’ g (CI 95%)**	0.687 (0.446–0.928)	0.743 (0.497–0.989)	0.712 (0.507–0.917)	0.663 (0.405–0.921)	0.651 (0.356–0.946)	0.407 (0.080–0.734)	0.802 (0.514–1.090)
***p*-value**	<0.001	<0.001	<0.001	<0.001	<0.001	0.015	<0.001

*CI* confidence interval, df degrees of freedom, *NDC* neurodevelopmental condition, *n* number of studies, *k* number of outcomes, *IDD* intellectual disability disorder, *ADHD* attention-deficit hyperactivity disorder.

##### Type of Measure

The type of measure used had a significant moderating effect for both attention (*n* = 49, *k* = 251, *Q*_(df=2)_ = 21.49, *p* < 0.001) and EF (*n* = 64, *k* = 368, *Q*_(df=1)_ = 107.38, *p* < 0.001), which resulted from larger effect sizes found using informant ratings compared to performance-based and/or physiological measures. Relative to the neurotypical group, the NDC group demonstrated more attention and EF delays on informant-based rating measures, compared to performance-based measures. There was evidence of overall attention and EF delays in the NDC group across both performance-based and informant-based measures. However, there were no differences in attention delays when measured using physiological measures, and not enough data to compare differences in EF delays when using physiological measures (Supplementary Tables 5 and 6).

### Qualitative analysis

**Fig. 4:**
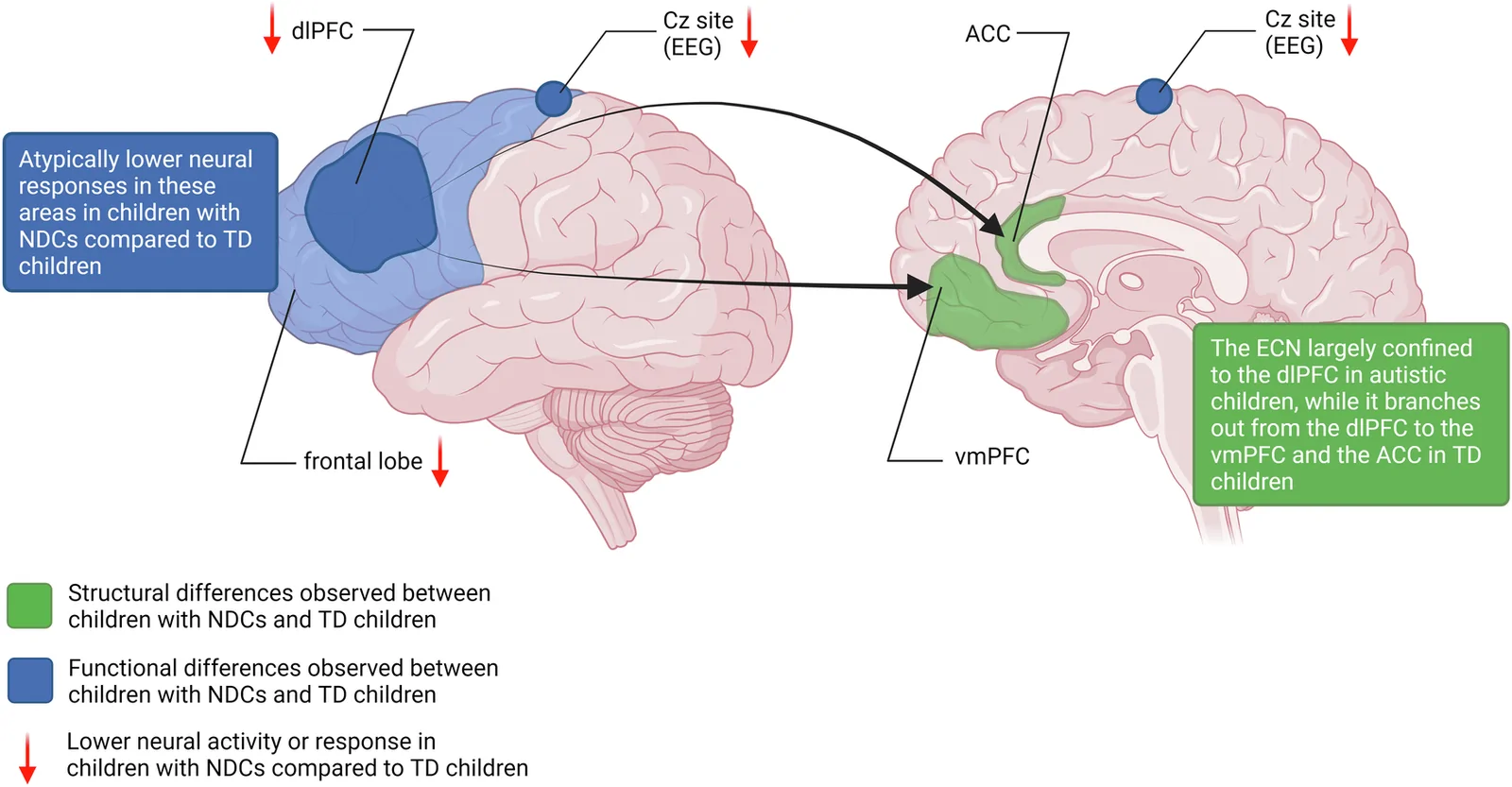
Early structural and functional brain divergence in children with NDCs. Structural brain divergence (in green) is captured by the limited volume and extent of the ECN in NDC children (specifically in autistic children) [35, 37], such that their ECN is largely confined to the dlPFC, while the ECN in neurotypical children branches from the dlPFC to the vmPFC and the ACC. Regions of divergent brain function (in blue) in children with NDCs include the dlPFC, the frontal lobe, and the Cz site (EEG), whereby children with NDCs (specifically children with autism or ADHD) show lower neural activity or response in these areas compared to neurotypical children either at resting state [38] or during the completion of an attention task [36, 37]. The marked regions are close approximations of the intended regions. Please also note one study that found no significant group differences was not included in this figure [39]. ACC anterior cingulate cortex, Cz site, midline central, dlPFC dorsolateral PFC, ECN executive control network, EEG electroencephalogram, NDC neurodevelopmental condition, PFC prefrontal cortex, vmPFC ventromedial PFC, dlPFC dorsolateral PFC.

Five studies adopted various brain-imaging techniques to explore the structural or functional brain development associated with attention or EF in children with NDCs (Supplementary Table 7). Four of these studies revealed both structural [35] and functional [36–38] brain divergences in the PFC and its associated regions in preschool children with autism or ADHD compared to their neurotypical peers (Supplementary Results 2). In contrast to this, the frontal activity of infants at risk for autism and/or ADHD while watching a non-social video was not significantly different to neurotypical infants [39]. An illustration of key findings is presented in Fig. 4.

## Discussion

This review reveals that children with NDCs show attention and EF delays in the first 5 years of life, with small and moderate effect sizes of delay, respectively. Delays were also observed when EF was analysed across its separate components. Moreover, there was evidence of increasing attention and EF impairments as children age. Delays could not be identified in infancy, began to emerge in toddlerhood and increased in size during the preschool period. The NDC groups showed EF delays, reflecting the transdiagnostic nature of this delay to NDCs. Interestingly, children with ADHD and communication disorders showed the greatest attention impairments compared to other NDCs. Across all types of measures, larger delays were observed from informant ratings compared to performance-based measures. Finally, there was evidence of atypical structure and function in the PFC and its associated regions in children with NDCs, which appear to be related to the observed attention and EF delays.

A pivotal finding of this review is that, overall, there was no evidence that attention and EF delays could be identified in the first year of life in NDCs, when compared to controls. While the PFC is an important neural underpinning of EF, it is a much broader system of connectivity both within the PFC and between the PFC and other cortical, subcortical, and limbic regions that subserve EF [26]. Although the PFC develops extensively in infancy [24, 25], the broader EF network that centres around the PFC remains largely premature and characterised by minimal development during the first year of life [40, 41]. While such brain maturation processes may be important to the emergence of NDCs, pre-emptive intervention approaches in the first year of life may not be able to use attention or EF markers to warrant targeted intervention. In contrast to this, small delays of both attention and EF began to emerge in toddlerhood and were found to increase further into the preschool period. This finding highlights an opportunity to reduce a widening delay with targeted interventions in toddlerhood. Notably, we found that studies with a high-risk sample tended to include younger children (typically infants) compared to studies where a clinically diagnosed NDC group was included. This is likely due to infants being able to be classified as high-risk based on familial history or exposure to identified risk factors, prior to the age at which a clinical diagnosis is typically made.

Early EF delays in NDCs were also found to occur broadly across most EF components, which parallel results from older children and adults [10, 15]. While different EF components are known to be characterised by distinct cognitive processes [1, 19], these components are mediated by a common neural circuitry that centres around the PFC [26, 42]. Following from this, it seems inevitable that the early neural divergence in children with NDCs broadly affects most EF components. These findings highlight the potential to conduct transdiagnostic intervention-based research in NDCs, targeting attention and executive function delays. Such transdiagnostic intervention approaches are often accepted as reflecting the realities of clinical practice, but they rarely make their way into clinical trials of therapeutics. As a result, there is currently no evidence of an effective transdiagnostic approach to improve attention or executive function delays early in life. Evidence from this study highlights the urgent need to develop frameworks and methodologies that can better capture the assessment and support needs of NDCs more broadly to improve detection and intervention opportunities [17].

The EF delays across most NDC groups support EF as a critical transdiagnostic feature of NDCs. While the majority of NDC groups also showed attention challenges, the nature and extent of these delays differed across different NDCs. Children with ADHD showed evidence of moderate attentional delays, consistent with the core diagnostic criteria of ADHD [43]. Children with communication disorders also showed a moderate attentional impairment, which aligns well with the emerging evidence that attentional deficits may be involved in the aetiology of linguistic deficits in communication disorders [44]. In contrast, while the effect of attentional delay in autistic children was not statistically significant, children in the high-risk autism group showed evidence of small attentional delays. Large population-based cohort studies have shown infants at high familial risk for autism are also at elevated risk of developing other NDCs like ADHD, learning and coordination disorders, intellectual disability, and tic disorders [45, 46]. This may explain why an attentional delay was observed in the high-risk autism group and opens the possibility that this delay may indicate a marker for other NDCs. These findings further emphasise a need for prospective studies that can track transdiagnostic markers of early neurodevelopmental delays [14, 47]. Finally, children with genetic conditions showed evidence of small attentional delays, which may be explained by the heterogeneity of the conditions combined under genetic conditions (e.g., Down’s syndrome, William’s syndrome). While there were not enough studies to conduct separate analyses between genetic conditions, this is an area for future investigation. Overall, while our results support a transdiagnostic approach to addressing EF and attention delays, extending on previous findings in older cohorts [15], the pattern and extent of attention delay may be informative to ADHD and communication-based NDCs.

Consistently larger attention and EF impairments were found when using informant ratings compared to performance-based or physiological measures. Performance-based measures capture the efficiency of the EF processes that are adopted during the completion of a goal-directed, structured task, while informant ratings provide ecologically valid evaluations of the individual’s ability to pursue goals in their everyday life [27]. Interestingly, no evidence of delays was found when attention was indexed using physiological measures, however, this may be due to limited power, with only 3 studies included in this analysis.

There was converging evidence from studies using various brain-imaging techniques, suggesting that attention and EF delays in preschool children with NDCs occur alongside atypical brain structure and function, specifically in and around the PFC [35–38]. These findings are consistent with the divergent neural underpinnings of attention and EF found in older children and adults with NDCs, and provides evidence that this neurodivergence begins to occur as early as preschool age [48–50].

Taken together, findings highlight an opportunity to apply attention and EF markers to prospectively detect important delays in transdiagnostic NDC cohorts. Attention and EF markers may have potential in general screening in practice for NDCs, but they appear less useful for differentiating specific NDCs – a task left for further neurodevelopmental assessment. Findings also have relevance for understanding the early emergence of NDCs and contribute to a broader transdiagnostic science of child development.

### Limitations

This review was limited by the currently available evidence (both published and unpublished), particularly for the younger age groups (infancy) and under-researched NDCs (e.g., cerebral palsy). While many of the included studies controlled for Intelligence/ Developmental Quotients (IQ/DQ), we did not include these variables as a moderator in our analysis. IQ/DQ delays are an inherent characteristic of many NDCs and introducing these variables as moderators may produce erroneous results [51]. Finally, while we sought to include unpublished datasets and the egger’s tests did not support evidence of publication bias, we cannot rule out the possibility of publication biases.

## Conclusion

In conclusion, this review has found that attention and EF delays in individuals with NDCs emerge prior to the age of 5, such that delays that are difficult to detect in infancy, begin to manifest in toddlerhood, and are further pronounced into the preschool years. These findings together contend that transdiagnostic detection and intervention approaches from toddlerhood are feasible and imperative to reduce lifelong impacts. Early intervention implemented at this period of substantial neural plasticity may ensure a more robust developmental foundation for children with NDCs. This could ultimately mitigate the lifelong personal, family, and societal costs that arise from delayed cognitive development.

## Supplementary information

Supplementary Materials

## Data Availability

The data used to undertake this meta-analysis are freely available at https://github.com/CarterSunUSYD/EF_attention_first5years_metaanalysis.git.

## Code availability

The R codes used for this meta-analysis are freely available at https://github.com/CarterSunUSYD/EF_attention_first5years_metaanalysis.git.
