# Supplementary material for: Attention and executive delays in early childhood: a meta-analysis of neurodevelopmental conditions
Source: Mol Psychiatry. 2024 Nov 3;30(5):1906–14. doi: 10.1038/s41380-024-02802-3 (PMC12015172; doi:10.1038/s41380-024-02802-3)
Supplement: Supplementary file 1 — Supplementary Materials [file 41380_2024_2802_MOESM1_ESM.docx]

**Supplementary Materials**

**Supplementary Table 1.** Search Strategy

**Supplementary Methods 1.** Eligibility Criteria

**Supplementary Methods 2.** Selection and Data Extraction Process

**Supplementary Methods 3.** Study Variables

**Supplementary Figure 1.** PRISMA Flowchart of Study Selection

**Supplementary Table 2.** Individual Studies in Meta-analysis

**Supplementary Table 3.** List of Excluded Outcomes at Statistical Analysis and Reasons for Exclusion

**Supplementary Figure 2.** Forest plot for Attention Studies.

**Supplementary Figure 3.** Forest plots for Executive Function separated by domains.

**Supplementary Results 1.** Bayesian Model Meta-analysis

**Supplementary Table 4.** Attention, Executive Function and Executive Function Components between Children with Neurodevelopmental Conditions Compared to Neurotypical Children

**Supplementary Table 5.** Moderator Analyses for Attention

**Supplementary Table 6.** Moderator Analyses for Executive Function

**Supplementary Table 7.** Individual Studies in the Qualitative Analysis

**Supplementary Results 2.** Qualitative Analysis

**References**

**Supplementary Table 1.** Search Strategy

| PsycINFO via Ovid |
| --- |
| 1 exp Neurodevelopmental Disorders/ 236410  2 exp Fetal Alcohol Syndrome/ 2100  3 exp Language Disorders/ 41463  4 exp Tourette Syndrome/ 3442  5 exp Dyspraxia/ 1070  6 exp Cerebral Palsy/ 6320  7 exp Fragile X Syndrome/ 2035  8 exp Williams Syndrome/ 1113  9 exp Prader Willi Syndrome/ 600  10 exp Rett Syndrome/ 975  11 exp Turners Syndrome/ 395  12 Angelman syndrome.mp. 485  13 happy puppet syndrome.mp. 3  14 Smith-Magenis syndrome.mp. 129  15 DiGeorge syndrome.mp. 346  16 velocardiofacial syndrome.mp. 179  17 22q11 deletion syndrome.mp. 172  18 Tuberous sclerosis.mp. 759  19 1 or 2 or 3 or 4 or 5 or 6 or 7 or 8 or 9 or 10 or 11 or 12 or 13 or 14 or 15 or 16 or 17 or 18 248136  20 exp Executive Function/ 23777  21 exp Attention/ 81648  22 working memory.mp. or exp Short Term Memory/ 50325  23 exp Response Inhibition/ 3935  24 exp Heart Rate/ 14408  25 exp Pupil Dilation/ 1299  26 attention.mp. 350388  27 24 and 26 1363  28 25 and 26 322  29 20 or 21 or 22 or 23 or 27 or 28 146839  30 19 and 29 12999  31 limit 30 to (peer reviewed journal and human and english language and (120 neonatal <birth to age 1 mo> or 140 infancy <2 to 23 mo> or 160 preschool age <age 2 to 5 yrs>)) |
| Medline via Ovid |
| 1 exp Neurodevelopmental Disorders/ 209140  2 exp Fetal Alcohol Spectrum Disorders/ 4668  3 exp Cerebral Palsy/ 23621  4 exp Fragile X Syndrome/ 5651  5 exp Williams Syndrome/ 1904  6 exp Prader-Willi Syndrome/ 3276  7 exp Rett Syndrome/ 2937  8 exp Turner Syndrome/ 7893  9 exp Angelman Syndrome/ 1377  10 exp Smith-Magenis Syndrome/ 239  11 exp DiGeorge Syndrome/ 2542  12 exp 22q11 Deletion Syndrome/ 2757  13 exp Tuberous Sclerosis/ 6732  14 1 or 2 or 3 or 4 or 5 or 6 or 7 or 8 or 9 or 10 or 11 or 12 or 13 262169  15 exp Executive Function/ 19542  16 exp Attention/ 86716  17 working memory.mp. or exp Memory, Short-Term/ 51017  18 exp Heart Rate/ 181084  19 pupil dilation.mp. 1680  20 attention.mp. 599506  21 response inhibition.mp. 5511  22 18 and 20 3038  23 19 and 20 327  24 15 or 16 or 17 or 21 or 22 or 23 148488  25 14 and 24 17102  26 limit 25 to (english language and humans and ("infant (1 to 23 months)" or "preschool child (2 to 5 years)") and journal article) |
| Embase via Ovid |
| 1 exp autism/ 95601  2 exp attention deficit disorder/ 8793  3 exp fetal alcohol syndrome/ 7747  4 exp intellectual impairment/ 635589  5 exp Gilles de la Tourette syndrome/ 9442  6 exp developmental delay/ 16971  7 exp communication disorder/ 89058  8 exp fluency disorder/ 1376  9 exp speech sound disorder/ 1180  10 exp learning disorder/ 42286  11 exp motor dysfunction/ 973304  12 exp developmental coordination disorder/ 1972  13 exp cerebral palsy/ 46922  14 exp fragile X syndrome/ 9990  15 exp Williams Beuren syndrome/ 3857  16 exp Prader Willi syndrome/ 6779  17 exp Rett syndrome/ 6237  18 exp Turner syndrome/ 13011  19 exp happy puppet syndrome/ 3279  20 exp Smith Magenis syndrome/ 741  21 exp DiGeorge syndrome/ 4262  22 exp chromosome deletion 22q11/ 2248  23 exp tuberous sclerosis/ 13384  24 exp Down syndrome/ 39997  25 exp velocardiofacial syndrome/ 1157  26 1 or 2 or 3 or 4 or 5 or 6 or 7 or 8 or 9 or 10 or 11 or 12 or 13 or 14 or 15 or 16 or 17 or 18 or 19 or 20 or 21 or 22 or 23 or 24 or 25 1690398  27 exp executive function/ 56185  28 exp attention/ 354701  29 exp working memory/ 51593  30 exp heart rate/ 314365  31 pupil dilation.mp. 2124  32 attention.mp. 788309  33 response inhibition.mp. 7277  34 30 and 32 5880  35 31 and 32 353  36 27 or 28 or 29 or 33 or 34 or 35 442134  37 26 and 36 62530  38 limit 37 to (human and english language and embase and journal and (infant <to one year> or preschool child <1 to 6 years>)) |

Please note searches were limited to peer-reviewed journals, English language, humans, and age range (0 to 5 years old). No restrictions were placed on the date of publication.

**Supplementary Methods 1.** Eligibility Criteria

A broad definition of NDCs was adopted, which encompasses the NDCs included in the DSM-5-TR and conditions that impair neurodevelopment through a known genetic or acquired aetiology – i.e., genetic disorders and cerebral palsy.(1) High-risk NDC was either defined by familial risk (i.e., family member diagnosed with an NDC) or exposure to a known risk factor (e.g., prenatal alcohol exposure in FASD). Children at risk for NDCs were included considering that reliable diagnoses cannot be given prior to a certain age, depending on the NDC diagnosis.

Studies with a non-clinical population (e.g., high NDC-like traits) were excluded. Studies where all participants had a comorbid medical condition and/or a neurological disorder were excluded.

Authors were contacted when studies did not report sufficient information for effect size calculation. Studies were excluded if the necessary information could not be obtained. In cases of considerable sample overlap between studies, the study that measured and/or reported the most information that satisfied the eligibility criteria was included.

**Supplementary Methods 2.** Selection and Data Extraction Process

Data were extracted using a predetermined data extraction form, which was developed to collect the data required for the meta-analyses (e.g., group attention and executive functioning (EF) scores as mean and standard deviation scores), moderator analyses (e.g., age group, gender distribution, NDC diagnosis and type of measure), and qualitative analysis.

**Supplementary Methods 3.** Study Variables

When EF outcomes were assessed using performance-based measures, they were coded into ‘simple working memory’, ‘complex working memory’, ‘simple response inhibition’, ‘complex response inhibition’, ‘response shifting’, and ‘attention shifting’ based on the categorisation and definitions proposed by Garon et al. (2008)(2). ‘Higher-order EF’ was included to this coding scheme as an additional domain to code for measures of planning and fluency.

The two EF questionnaires identified were the Behavior Rating Inventory of Executive Function Preschool (BRIEF-P)(3) and the temperamental questionnaires developed by Putnam et al. (2001)(4). Given the conceptual overlap of two subscales in the temperamental questionnaires (i.e., impulsivity and inhibitory control) with the inhibit subscale of the BRIEF-P, the three subscales were analysed under a single subscale ‘inhibit’.

Four variables were selected for moderator analysis: age group, gender distribution, NDC diagnosis and type of measure. First, attention and EF differences were analysed across three age groups: infancy (0 – 1 year old), toddlerhood (1 – 3 years old) and the preschool period (3 – 5 years old), as early EF development appears to be demarcated by these three developmental phases.(2, 5, 6) Gender distribution was coded as the percentage of boys in the NDC group and analysed as a continuous moderator, as maturation rates tend to differ between genders.(7) The transdiagnostic nature of attention and EF delays were explored by including NDC diagnosis as a moderator. Finally, the effect of different types of measures was investigated.

**Identification of studies via other methods**

**Identification of studies via databases and registers**

Records identified from:

Unpublished data requested from authors (n = 1)

Records removed *before screening*:

Duplicate records removed (n = 578)

Records identified from*:

Databases (n = 4916)

**Identification**

Records screened

(n = 4338)

Records excluded

(n = 3683)

Reports not retrieved

(n = 0)

Reports sought for retrieval

(n = 1)

Reports sought for retrieval

(n = 653)

Reports not retrieved

(n = 3)

**Screening**

Reports excluded:

Study did not satisfy eligibility criteria (n = 444)

Ineligible outcome, diagnosis, age, study design, type of study or language

Insufficient data (n = 82)

Study with overlapping sample (n = 9)

Reports assessed for eligibility

(n = 1)

Reports assessed for eligibility

(n = 650)

Studies included in main quantitative analysis (n = 111)

Studies included in qualitative analysis (n = 5)

**Included**

**Supplementary Figure 1.** PRISMA Flowchart of Study Selection

**Supplementary Table 2.** Individual Studies Included in Meta-analysis

| **Author & year** | **Diagnostic Group** | **Control Sample size** | **Outcome** | **Hedge’s g, (variance)** | **Age Years (Mean)** | **Gender Distribution (% of Males)** | **Quality Assessment** |
| --- | --- | --- | --- | --- | --- | --- | --- |
| Alesi et al. (2018)(8) | Motor Disorders (n = 18) | 18 | EF: Simple Working Memory  Complex Working Memory  Higher-order EF  Simple Response Inhibition  Complex Response Inhibition | 0.71 (0.12) | 4.6 | 50 | Good |
| Aljahlan et al. (2019)(9) | Communication Disorders (n = 25) | 25 | Attention | 0.65 (0.09) | 4.22 | 69 | Good |
| Anderson et al. (2010)(10) | Communication Disorders (n = 9) | 14 | Attention | 0.42 (0.19) | 4.28 | 67 | Good |
|  |  |  | EF: Simple Working Memory  CBQ Impulsivity  CBQ Inhibitory Control | 1.11 (0.21) |  |  |  |
| Anderson et al. (2017)(11) | Communication Disorders (n = 41) | 41 | EF: Complex Response Inhibition  CBQ Inhibitory Control | 0.37 (0.05) | 4.36 | 71 | Good |
| Smith et al. (unpublished)(12) | Autism (n = 33) | 35 | Attention | -0.16 (0.06) | 2.16 | 78.79 | Good |
| Bavin et al. (2005)(13) | Communication Disorders (n = 21) | 21 | EF: Simple Working Memory  Complex Working Memory | 0.44 (0.10) | 4.5 | 52 | Good |
| Bazelmans et al. (2019)(14) | Autism (n = 71) | 66 | Attention | 0.18 (0.03) | 3.19 | 90.14 | Good |
|  |  |  | BRIEF Global EF | 1.39 (0.04) |  |  |  |
| Bradshaw et al. (2020)(15) | High-risk Autism (n = 41) | 39 | Attention | 0.20 (0.05) | 0.02 | 56 | Good |
| Brown et al. (2003)(16) | Genetic Disorders (n = 13), Genetic Disorders (n = 19) | 17 | Attention | 0.44 (0.14) | 2.42, 2.42 | Not reported | Good |
| Bryson et al. (2018)*(17) | High-risk Autism (n = 47), Autism (n = 9) | 53 | Attention | 0.02 (0.09) | 0.5, 0.5 | 55.22, 43.75 | Good |
| Byrne et al. (2003)(18) | ADHD (n = 24) | 24 | Attention | 0.72 (0.09) | 4.77 | 84 | Good |
|  |  |  | EF: Simple Response Inhibition  Complex Response Inhibition | 0.64 (0.09) |  |  |  |
| Byrne et al. (1998)(19) | ADHD (n = 13) | 13 | Attention | 0.59 (0.17) | 4.62 | 84.62 | Good |
|  |  |  | EF: Simple Response Inhibition | 0.54 (0.16) |  |  |  |
| Cak et al. (2017)(20) | ADHD (n = 21) | 52 | Attention | 0.49 (0.07) | 4.84 | 85.71 | Good |
|  |  |  | EF: Complex Response Inhibition  BRIEF -P Inhibit  BRIEF-P Shift  BRIEF-P Emotional Control  BRIEF-P Working Memory  BRIEF-P Plan/Organize  BRIEF-P Inhibitory Self-Control  BRIEF-P Flexibility  BRIEF-P Emergent Metacognition  BRIEF-P Global Executive Function | 1.57 (0.10) |  |  |  |
| Cardon et al. (2012)(21) | Autism (n = 8) | 9 | Attention | 0.99 (0.27) | 3.46 | 87.5 | Fair |
| Chawarska et al. (2003)(22) | Autism (n = 10) | 16 | Attention | -0.02 (0.16) | 2.5 | 80 | Good |
| Cheung et al. (2018)(23) | High-risk Autism (n = 64), High-risk Autism (n = 31), Autism (n = 17) | 27 | Attention | -0.48 (0.08) | 0.76, 0.75, 0.736 | 43.75, 62.5, 88.24 | Good |
| Clark et al. (2015)(24) | Communication Disorders (n = 82) | 120 | Attention | -0.16 (0.02) | 3.89 | 79.27 | Good |
| Constantinou et al. (2007)(25) | Cerebral Palsy (n = 14) | 54 | Attention | 0.73 (0.09) | .69 | 56 | Fair |
| Dawson et al. (2002)(26) | Autism (n = 72), Mixed NDCs (n = 34) | 39 | EF: Response Shifting  Complex Working Memory | -0.01 (0.05) | 3.63, 3.73 | 83.33, 52.94 | Good |
| DeWolfe et al 1999*(27) | ADHD (n = 25) | 25 | Attention | 0.52 (0.08) | 4.82 | 84 | Good |
|  |  |  | EF: Simple Response Inhibition  Complex Response Inhibition | 0.54 (0.08) |  |  |  |
| DeWolfe et al 2000*(28) | ADHD (n = 25) | 25 | Attention | 0.69 (0.09) | 4.82 | 84 | Good |
|  |  |  | EF: Simple Response Inhibition | 0.32 (0.08) |  |  |  |
| Edmunds et al 2022(29) | Autism (n = 65) | 54 | EF: Complex Working Memory | 0.69 (0.06) | 4.51 | 81.54 | Fair |
| Eichorn et al 2018(30) | Communication Disorders (n = 16) | 30 | EF: Attention Shifting | 0.29 (0.10) | 4.14 | 75 | Good |
| Elsabbagh et al 2009*(31) | High-risk Autism (n = 16) | 16 | Attention | 0.39 (0.13) | 0.83 | 75 | Good |
| Elsabbagh et al 2013*(32) | Autism (n = 16), high-risk Autism (n = 12) | 48 | Attention | -0.24 (0.09) | 0.63. 0.61 | 64.71, 25 | Good |
| Ezpeleta et al 2015(33) | ADHD (n = 23), comorbid ADHD and oppositional defiant disorder (n = 10) | 538 | Attention | 0.39 (0.07) | 3.74, 3.69 | 73.9, 40 | Good |
|  |  |  | EF: Complex Response Inhibition  BRIEF -P Inhibit  BRIEF-P Shift  BRIEF-P Emotional Control  BRIEF-P Working Memory  BRIEF-P Plan/Organize  BRIEF-P Inhibitory Self-Control  BRIEF-P Flexibility  BRIEF-P Emergent Metacognition  BRIEF-P Global Executive Function | 0.31 (0.07) |  |  |  |
| Fanning et al 2018(34) | Autism (n = 26), Genetic Disorders (n = 18) | 19 | EF: Response Shifting | 0.50 (0.10) | 3.79, 4.15 | 84.62, 55.56 | Good |
| Farzin et al. 2010(35) | Genetic Disorders (n = 32) | 34 | Attention | 0.81 (0.07) | 2.29 | 84.38 | Good |
|  |  |  | EF: Simple Working Memory | 0.61 (0.07) |  |  |  |
| Ference et al 2013(36) | High-risk Autism (n = 26) | 30 | Attention | 0.11 (0.07) | 0.42 | 50 | Good |
| Waring et al 2018*(37) | Communication Disorders (n = 16) | 50 | EF: Simple Working Memory  Complex Working Memory | 0.58 (0.09) | 4.08 | 68.75 | Good |
| Foltz et al 2015(38) | Communication Disorders (n = 8) | 8 | EF: Simple Working Memory | 1.15 (0.29) | 4.83 | 75 | Good |
| Freire et al 2020(39) | Cerebral Palsy (n = 14) | 14 | EF: Simple Working Memory  Complex Working Memory  Response Shifting  Complex Response Inhibition | 0.47 (0.15) | 4.97 | 64.3 | Good |
| Fuglestad et al 2015(40) | Fetal Alcohol Spectrum (n = 39) | 50 | EF: Response Shifting  Simple Response Inhibition | 0.99 (0.05) | 4.4 | 33 | Good |
| Garon et al 2018*(41) | Autism (n = 34) | 255 | EF: Attention Shifting  Complex Working Memory  Complex Response Inhibition | 0.78 (0.03) | 4.57 | 79.41 | Good |
| Gooch et al 2014(42) | Communication Disorders (n = 25), High-risk Communication Disorders (n = 83), Communication Disorder (n = 27) | 68 | Attention | 0.83 (0.05) | 3.63, 3.76, 3.76 | 69, 58, 72 | Good |
|  |  |  | EF: Complex Response Inhibition | 0.58 (0.07) |  |  |  |
| Guiberson et al 2020(43) | Communication Disorders (n = 37) | 93 | EF: Simple Working Memory | 0.62 (0.04) | 4.5 | 51.35 | Good |
| Holmboe et al 2010(44) | High-risk Autism (n = 31) | 33 | Attention | 0.43 (0.06) | 0.83 | 58.06 | Good |
|  |  |  | EF: Simple Response Inhibition | -0.44 (0.09) |  |  |  |
| Jahromi et al 2013*(45) | Autism (n = 20) | 20 | EF: Complex Response Inhibition  CBQ Effortful Self-Control  BRIEF-P Inhibitory Self-Control | 1.15 (0.12) | 4.91 | 90 | Good |
| Jahromi et al 2019*(46) | Autism (n = 18) | 20 | EF: Simple Response Inhibition  Complex Response Inhibition  CBQ Effortful Control | 0.96 (0.12) | 4.8 | Not Reported | Good |
| St John et al 2016*(47) | Autism (n = 101), high-risk Autism (n = 23) | 50 | EF: Response Shifting | -0.13 (0.05) | 1.04, 1.04 | 73.9, 56.4 | Good |
| Joyce et al 2020(48) | Genetic Disorders (n = 75) | 460 | EF: BRIEF -P Inhibit  BRIEF-P Shift  BRIEF-P Emotional Control  BRIEF-P Working Memory  BRIEF-P Plan/Organize  BRIEF-P Inhibitory Self-Control  BRIEF-P Flexibility  BRIEF-P Emergent Metacognition  BRIEF-P Global Executive Function | 2.01 (0.02) | 4.74 | 61.33 | Good |
| Coles et al 2021(49) | High-risk Fetal Alcohol Spectrum (n = 109) | 174 | Attention | 0.21 (0.02) | 4.02 | 48.7 | Good |
|  |  |  | EF: Simple Working Memory  Simple Response Inhibition  Response Shifting  Complex Working Memory  Complex Response Inhibition | 0.07 (0.02) |  |  |  |
| Kalliontzi et al 2022(50) | Communication Disorders (n = 53) | 63 | EF: Attention Shifting  Complex Working Memory  Complex Response Inhibition | 0.24 (0.04) | 4.45 | 68 | Good |
| Kapa et al 2020*(51) | Communication Disorders (n = 41) | 41 | EF: Attention Shifting  Simple Working Memory  Complex Working Memory  Complex Response Inhibition | 4.88 (0.24) | 4.95 | 63.41 | Good |
| Kapa et al 2017*(52) | Communication Disorders (n = 26) | 26 | EF: Attention Shifting  Complex Working Memory  Complex Response Inhibition | 0.66 (0.08) | 4.93 | 50 | Good |
| Kimhi et al 2014(53) | Autism (n = 29) | 30 | EF: Attention Shifting  Higher-order EF | 0.53 (0.07) | 4.95 | 86.21 | Good |
| Krakow et al 1982*(54) | Genetic Disorders (n = 16) | 40 | Attention | -0.06 (0.10) | 2.42 | 62.5 | Fair |
| Krakow et al 1983*(55) | Genetic Disorders (n = 17), Intellectual Developmental Disorders (n = 14) | 40 | Attention | 0.03 (0.12) | 2.4, 2.17 | 86, 59 | Fair |
| Kuhl et al 2021(56) | ADHD (n = 61), comorbid ADHD and oppositional defiant disorder (n = 52) | 58 | Attention |  | 4.6, 4.51 | 79, 79 | Good |
|  |  |  | EF: Simple Response Inhibition  Complex Working Memory  Complex Response Inhibition | 0.81 (0.04) |  |  |  |
| Loveall et al 2017(57) | Genetic Disorders (n = 22) | 460 | EF: BRIEF -P Inhibit  BRIEF-P Shift  BRIEF-P Emotional Control  BRIEF-P Working Memory  BRIEF-P Plan/Organize  BRIEF-P Inhibitory Self-Control  BRIEF-P Flexibility  BRIEF-P Emergent Metacognition  BRIEF-P Global Executive Function | 0.81 (0.05) | 3.6 | 40.9 | Fair |
| Macari et al 2017(58) | Autism (n = 165), Intellectual Developmental Disorders (n = 58) | 92 | EF: TBAQ Effortful Control  TBAQ Attentional Focusing  TBAQ Attentional Shifting  TBAQ Inhibitory Control | 0.91 (0.02) | 2.21, 2.2 | 81.80, 77.5 | Good |
| Maestro et al 2002*(59) | Autism (n = 15) | 15 | Attention | 0.23 (0.14) | 0-6 Months | 66.67 | Good |
| Maestro et al 2005*(60) | Autism (n = 15) | 13 | Attention | -0.07 (0.14) | 0-6 Months | 73.33 | Good |
| Mahone et al 2007*(61) | ADHD (n = 25) | 25 | EF: BRIEF -P Inhibit  BRIEF-P Shift  BRIEF-P Emotional Control  BRIEF-P Working Memory  BRIEF-P Plan/Organize  BRIEF-P Inhibitory Self-Control  BRIEF-P Flexibility  BRIEF-P Emergent Metacognition  BRIEF-P Global Executive Function | 1.94 (0.12) | 4.86 | 80 | Good |
| Mahone et al 2005*(62) | ADHD (n = 40) | 40 | Attention | 0.77 (0.05) | 5 | 85 | Good |
|  |  |  | EF: Simple Response Inhibition  Complex Working Memory  Complex Response Inhibition | 0.54 (0.05) |  |  |  |
| Ntourou et al 2018(63) | Communication Disorders (n = 75) | 75 | EF: Complex Response Inhibition  BRIEF -P Inhibit  BRIEF-P Shift  BRIEF-P Emotional Control  BRIEF-P Working Memory  BRIEF-P Plan/Organize  BRIEF-P Inhibitory Self-Control  BRIEF-P Flexibility  BRIEF-P Emergent Metacognition  BRIEF-P Global Executive Function | 0.29 (0.03) | 4.10 | 69.33 | Good |
| Parhiala et al 2014(64) | Specific Learning Disorder (n = 39) | 131 | Attention | 0.41 (0.03) | 4 | 48.7 | Fair |
| Pellicano et al 2017(65) | Autism (n = 30) | 30 | EF Attention Shifting  Complex Working Memory  Complex Response Inhibition | 1.32 (0.08) | 4.44 | 90 | Good |
| Powell et al 2022(66) | Autism (n = 73), high-risk Autism (n = 33), Mixed NDCs (n = 35) | 28 | EF:  BRIEF-P emergent metacognition  BRIEF-P flexibility  BRIEF-P inhibitory self-control  BRIEF-P global executive function | 0.63 (0.06) | 3.28, 3.22, 3.25 | 80.8, 60.6, 54.3 | Good |
| Putra et al 2021(67) | Autism (n = 22) | 35 | Attention | 0.55 (0.08) | 4.6 | 72.7 | Fair |
|  |  |  | EF: Complex Response Inhibition | 0.15 (0.07) |  |  |  |
| Ratto et al 2020(68) | Autism (n = 55) | 460 | EF:  BRIEF -P Inhibit  BRIEF-P Shift  BRIEF-P Emotional Control  BRIEF-P Working Memory  BRIEF-P Plan/Organize  BRIEF-P Inhibitory Self-Control  BRIEF-P Flexibility  BRIEF-P Emergent Metacognition  BRIEF-P Global Executive Function | 1.87 (0.02) | 4.75 | 80 | Good |
| Roberts et al 2015(69) | Genetic Disorders (n = 13) | 13 | EF: Response Shifting  Simple Working Memory | 0.21 (0.16) | 4.34 | 52.9 | Fair |
| Sasson et al 2011(70) | Autism (n = 9) | 13 | Attention | -0.14 (0.19) | 3.6 | 88.9 | Good |
| Wittke et al 2013(71) | Communication Disorder (n = 19) | 19 | EF:  BRIEF-P Inhibitory Self-Control  BRIEF-P Flexibility  BRIEF-P Emergent Metacognition  BRIEF-P Global Executive Function | 0.97 (0.12) | 4.13 | 63.2 | Good |
| Schneider et al 2020(72) | ADHD (n = 49) | 35 | BRIEF-P Inhibit  BRIEF-P Plan/Organize  BRIEF-P Shift  BRIEF-P Emotional Control  BRIEF-P Working Memory | 1.46 (0.06) | 5 | 59.2 | Good |
| Schoemaker et al 2014*(73) | ADHD (n = 59), Comorbid ADHD + oppositional defiant disorder (n = 50) | 58 | EF:  Complex Working Memory  Complex Response Inhibition | 1.02 (0.04) | 4.59, 4.49 | 79.7, 82 | Good |
| Schwenk et al 2007(74) | Communication Disorders (n = 13) | 14 | Attention | 0.61 (0.16) | 3.93 | 55.56 | Good |
| Skogan et al 2015*(75) | ADHD (n = 104) | 117 | EF:  BRIEF -P Inhibit  BRIEF-P Shift  BRIEF-P Emotional Control  BRIEF-P Working Memory  BRIEF-P Plan/Organize  BRIEF-P Inhibitory Self-Control  BRIEF-P Flexibility  BRIEF-P Emergent Metacognition | 1.42 (0.02) | 3.48 | 63.5 | Good |
| Smith et al 2019(76) | Autism (n = 29) | 30 | Attention | 0.20 (0.07) | 2.23 | 96.55 | Good |
|  |  |  | EF:  Attention Shifting | 0.04 (0.07) |  |  |  |
| Waring et al 2017*(77) | Communication Disorders (n = 14) | 50 | EF:  Simple Working Memory  Complex Working Memory | 0.67 (0.09) | 4.33 | 78.57 | Good |
| Tonnsen et al 2018(78) | High-Risk Autism (n = 21) | 21 | Attention | 0.06 (0.10) | 0.82 | 80.95 | Good |
| Wang et al 2020(79) | Intellectual Developmental Disorders (n = 31), Autism (n = 48) | 36 | Attention | -0.21 (0.10) | 3.81, 3.19 | 61, 77 | Good |
| Vugs et al 2014*(80) | Communication Disorders (n = 58) | 58 | EF:  Simple Working Memory  Complex Working Memory | 1.39 (0.04) | 4.75 | 72.41 | Good |
| Barnes et al 2020(81) | Specific Learning Disorder (n = 164) | 286 | Attention | 0.64 (0.02) | 4.5 | 53.3 | Good |
|  |  |  | EF:  Simple Working Memory  Complex Response Inhibition | 0.33 (0.02) |  |  |  |
| Hernandez et al 2022(82) | ADHD (n = 53) | 47 | EF:  Attention Shifting  Global Executive Function  Complex response Inhibition | 1.13 (0.05) | 4.62 | 86.8 | Good |
| Noland et al 2010(83) | High-Risk Autism (n = 19) | 22 | Attention | -0.02 (0.10) | 0.55 | 73 | Fair |
|  |  |  | EF:  Simple Working Memory | -0.44 (0.10) |  |  |  |
| Pauli-Pott et al 2014(84) | High-risk ADHD (n = 30) | 30 | EF:  Simple Response Inhibition  Complex Response Inhibition | 0.26 (0.07) | 4.87 | 66.67 | Good |
| Ruskin et al 1994(85) | Genetic Disorders (n = 40) | 25 | Attention | 0.11 (0.07) | 1.89 | 45.24 | Good |
| Sacrey et al 2013(86) | High-risk Autism (n = 10), Autism (n = 10) | 10 | Attention | 0.07 (0.20) | 0.59, 0.71 | 30, 60 | Good |
| Scerif et al 2004*(87) | Genetic Disorders (n = 8), Genetic Disorders (n = 8) | 8 | EF:  Complex Response Inhibition | 0.86 (0.28) | 3.82, 3.63 | 50, 100 | Poor |
| Scerif et al 2007*(88) | Genetic Disorders (n = 13) | 13 | Attention | 0.61 (0.17) | 4.43 | 100 | Fair |
|  |  |  | EF:  Simple Response Inhibition  Complex Response Inhibition | 0.82 (0.17) |  |  |  |
| Schoemaker et al 2012*(89) | ADHD (n = 61), comorbid ADHD+ oppositional defiant disorder (n = 52) | 56 | Attention | 2.80 (0.07) | 4.6, 4.51 | 80.3, 82.7 | Good |
|  |  |  | EF:  Simple Response Inhibition  Complex Working Memory  Complex Response Inhibition | 0.70 (0.04) |  |  |  |
| Schworer et al 2022(90) | Genetic Disorders (n = 36) | 32 | Attention | 0.88 (0.06) | 1.05 | 47 | Fair |
|  |  |  | EF: higher-order EF | 0.64 (0.06) |  |  |  |
| Skogan et al 2014*(91) | ADHD (n = 150), comorbid ADHD + oppositional defiant disorder (n = 205) | 455 | EF:  Simple Working Memory  Simple Response Inhibition  Complex Working Memory | 0.21 (0.01) | 3.46, 3.475 | 53.3, 57.9 | Good |
| Snowling et al 2018(92) | Communication Disorders (n = 64), High-risk Communication Disorders (n = 90) | 74 | Attention | 0.59 (0.03) | 4.5 | Not Reported | Fair |
|  |  |  | EF: Simple Working memory  Complex Response Inhibition | 0.76 (0.03) |  |  |  |
| Sorensen et al 2016(93) | Cerebral Palsy (n = 14) | 460 | EF:  BRIEF-P Inhibitory Self-Control  BRIEF-P Flexibility  BRIEF-P Global Executive Function | 0.18 (0.08) | 3.25, | 50 | Good |
| Tonnsen et al 2015(94) | Genetic Disorders (n = 14) | 14 | Attention | 1.78 (0.21) | 4.05 | 100 | Fair |
|  |  |  | EF: Simple Response Inhibition | 1.05 (0.16) |  |  |  |
| Yerys et al 2007(95) | Autism (n = 16), Mixed NDCs (n = 17) | 15 | EF: Response Shifting | -0.30 (0.13) | 2.9, 2.96 | 83.33, 55.56 | Good |
| Onnivello et al 2022(96) | Genetic Disorders (n = 40) | 460 | EF:  BRIEF-P Inhibit  BRIEF-P Shift  BRIEF-P Emotional Control  BRIEF-P Working Memory  BRIEF-P Plan/Organize  BRIEF-P Inhibitory Self-Control  BRIEF-P Flexibility  BRIEF-P Emergent Metacognition  BRIEF-P Global Executive Functioning | 0.77 (0.03) | 4.76 | 62.5 | Fair |
| Precenzano et al 2017(97) | Autism (n = 8) | 15 | EF:  BRIEF-P Inhibit  BRIEF-P Shift  BRIEF-P Emotional Control  BRIEF-P Working Memory  BRIEF-P Plan/Organize  BRIEF-P Inhibitory Self-Control  BRIEF-P Flexibility  BRIEF-P Emergent Metacognition  BRIEF-P Global Executive Functioning | 1.20 (0.22) | 3.09 | 87.5 | Fair |
| Smithson et al 2013(98) | Autism (n = 39) | 460 | EF:  BRIEF-P Inhibit  BRIEF-P Shift  BRIEF-P Emotional Control  BRIEF-P Working Memory  BRIEF-P Plan/Organize  BRIEF-P Inhibitory Self-Control  BRIEF-P Flexibility  BRIEF-P Emergent Metacognition  BRIEF-P Global Executive Functioning | 1.49 (0.03) | 4.38 | 82.05 | Good |
| Watson 2012(99) | Autism (n = 22) | 15 | Attention | -0.32 (0.12) | 2.92 | 100 | Good |
| Wang 2023(100) | Autism (n = 24) | 24 | EF:  Simple Working Memory  Complex Response Inhibition | 1.15 (0.11) | 4.63 | 54.17 | Good |
| McClain et al 2022(101) | Autism (n = 24), Comorbid Autism + Intellectual Developmental Disorders (n = 40), Intellectual Developmental Disorders (n = 23) | 460 | EF:  BRIEF-P Inhibit  BRIEF-P Shift  BRIEF-P Emotional Control  BRIEF-P Working Memory  BRIEF-P Plan/Organize  BRIEF-P Inhibitory Self-Control  BRIEF-P Flexibility  BRIEF-P Emergent Metacognition  BRIEF-P Global Executive Functioning | 2.20 (0.04) | 4.09, 3.86, 3.77 | 87.5, 77.5, 78.26 | Fair |
| Rudling et al 2022(102) | High-risk Autism (n = 50), Autism (n = 18) | 31 | Attention | -0.69 (0.08) | 0.86, 0.85 | 40, 55.56 | Good |
| Scerif et al 2005*(103) | Genetic Disorders (n = 10) | 10 | EF: Simple Response Inhibition | -0.01 (0.20) | 2.99 | 100 | Fair |
| Zhang et al 2018(104) | ADHD (n = 163) | 63 | EF:  Simple Working Memory  Simple Response Inhibition  BRIEF-P Inhibit  BRIEF-P Shift  BRIEF-P Emotional Control  BRIEF-P Working Memory  BRIEF-P Plan/Organize  BRIEF-P Inhibitory Self-Control  BRIEF-P Flexibility  BRIEF-P Emergent Metacognition  BRIEF-P Global Executive Functioning | 0.57 (0.02) | 4.93 | 82.21 | Good |
| Zwaigenbaum et al 2005(105) | High-risk Autism (n = 25) | 25 | Attention | 0.27 (0.08) | 0.54 | Not Reported | Good |
| Ohmann et al 2022(106) | ADHD (n = 115) | 460 | EF:  BRIEF-P Inhibit  BRIEF-P Shift  BRIEF-P Emotional Control  BRIEF-P Working Memory  BRIEF-P Plan/Organize  BRIEF-P Inhibitory Self-Control  BRIEF-P Flexibility  BRIEF-P Emergent Metacognition  BRIEF-P Global Executive Functioning | 1.29 (0.01) | 4.3 | 79.13 | Good |
| Berger et al 2019(107) | Cerebral Palsy (n = 9) | 22 | Attention | -0.16 (0.16) | 1.17 | 66.67 | Good |
| Chu et al 2019(108) | Specific Learning Disorder (n = 14) | 35 | EF: Complex Response Inhibition | 0.79 (0.11) | 4.05 | 50 | Good |
| St John et al 2023*(109) | High-risk Autism (n = 45), High-risk Autism (n = 65) | 30, 25 | EF: Response Shifting | 0.59 (0.06) | 2.05, 2.04 | 0, 100 | Good |
| Vugs et al 2017*(110) | Communication Disorders (n = 30) | 33 | EF:  Simple Working memory  Complex Working Memory | 1.60 (0.09) | 4.56 | 70 | Good |
| Fitch et al 2019(111) | Autism (n = 22) | 26 | Attention | 0.83 (0.09) | 2.33 | 95.45 | Good |
| Afshar 2022(112) | Communication Disorders (n = 16), Communication Disorders (n = 15) | 18 | Attention | 1.86 (0.17) | 4.99, 4.87 | 80, 62.5 | Good |
|  |  |  | EF:  Attention Shifting  Simple Working Memory  Complex Response Inhibition | 3.14 (0.30) |  |  |  |
| Everaert et al 2023(113) | Genetic Disorders (n = 44) | 81 | Attention | 0.58 (0.04) | 4.9 | 57 | Good |
|  |  |  | EF:  Simple Working Memory  Complex Working Memory  Complex Response Inhibition | 0.31 (0.08) |  |  |  |
| Dimachkie Nunnally 2023(114) | Genetic Disorders (n = 41) | 460 | EF:  BRIEF-P Inhibit  BRIEF-P Shift  BRIEF-P Emotional Control  BRIEF-P Working Memory  BRIEF-P Plan/Organize  BRIEF-P Inhibitory Self-Control  BRIEF-P Flexibility  BRIEF-P Emergent Metacognition | 0.71 (0.03) | 3.25 | 49 | Good |
| Thistle et al 2022(115) | ADHD (n = 257) | 77 | EF:  Simple Working Memory  Simple Response Inhibition  BRIEF-P Inhibit  BRIEF-P Emotional Control  BRIEF-P Working Memory | 0.17 (0.02) | 3.48 | 44 | Good |
| Pouretemad et al 2022(116) | Autism (n = 15) | 15 | EF:  BRIEF-P Inhibit  BRIEF-P Shift  BRIEF-P Emotional Control  BRIEF-P Working Memory  BRIEF-P Plan/Organize  BRIEF-P Inhibitory Self-Control  BRIEF-P Flexibility  BRIEF-P Emergent Metacognition  BRIEF-P Global Executive Functioning | 1.63 (0.18) | 2.28 | 73.33 | Good |
| Webster et al 1997(117) | Communication Disorders (n = 15) | 15 | EF: Simple Working Memory | 1.26 (0.16) | 3.57 | Not Reported | Good |
| Stokes et al 2006(118) | Communication Disorders (n = 14) | 15 | EF: Simple Working Memory | 1.32 (0.17) | 4.92 | Not Reported | Fair |

Abbreviations: n, sample size; EF, executive function; CBQ, children behavior questionnaire; ADHD, attention-deficit hyperactivity disorder; BRIEF-P, behavior rating inventory of executive function preschool; NDC, neurodevelopmental condition; TBAQ, toddler behavior assessment questionnaire. *Studies were nested when performing meta-analysis as they reported on overlapping cohorts.

**Supplementary Table 3.** List of Excluded Outcomes at Statistical Analysis and Reasons for Exclusion

| **Author & Year** | **Title** | **Outcome** | **Exclusion reason** |
| --- | --- | --- | --- |
| Kapa et al 2020(51) | The Relationship Between Word Learning and Executive Function in Preschoolers With and Without Developmental Language Disorder | EF:  Complex Working Memory  Attention Shifting | Study identified as an outlier (Hedge’s g > 2), removed to ensure statistical integrity |
| Afshar et al 2022(119) | Executive Functions in Persian-Speaking Preschool Children with Speech Sound Disorders and Comparison with their Typically Developing Peers | EF:  Simple Working Memory  Attention Shifting, Complex Response Inhibition, Complex Working Memory, | Study identified as an outlier (Hedge’s g > 2), removed to ensure statistical integrity |
| McClain et al 2022(120) | Executive functioning skills in early childhood children with autism, intellectual disability, and co-occurring autism and intellectual disability | EF:  Global Executive Function | Study identified as an outlier (Hedge’s g > 2), removed to ensure statistical integrity |
| Kapa et al 2017(121) | Applying an integrative framework of executive function to preschoolers with specific language impairment | EF:  Attention Shifting, Complex Response Inhibition, Simple Working Memory, Complex Working Memory | Study identified as an outlier (Hedge’s g > 2), removed to ensure statistical integrity |
| Joyce et al 2020(48) | Obstructive sleep apnoea contributes to executive function impairment in young children with Down syndrome | EF  Global Executive Function | Study identified as an outlier (Hedge’s g > 2), removed to ensure statistical integrity |
| Schoemaker et al 2012(122) | Executive function deficits in preschool children with ADHD and DBD. | Attention  Attention Problems | Study identified as an outlier (Hedge’s g > 2), removed to ensure statistical integrity |
| Kuhl et al 2021(123) | Trajectories of Executive Functions and ADHD Symptoms in Preschoolers and the Role of Negative Parental Discipline. | Attention  Attention Problems | Study identified as an outlier (Hedge’s g > 2), removed to ensure statistical integrity |
| Hernandez et al 2022(82) | Individual differences in germ spreading behaviors among children with attention-deficit/hyperactivity disorder: The role of executive functioning | Attention  Attention Problems | Study identified as an outlier (Hedge’s g > 2), removed to ensure statistical integrity |

Abbreviations: EF, executive function

**
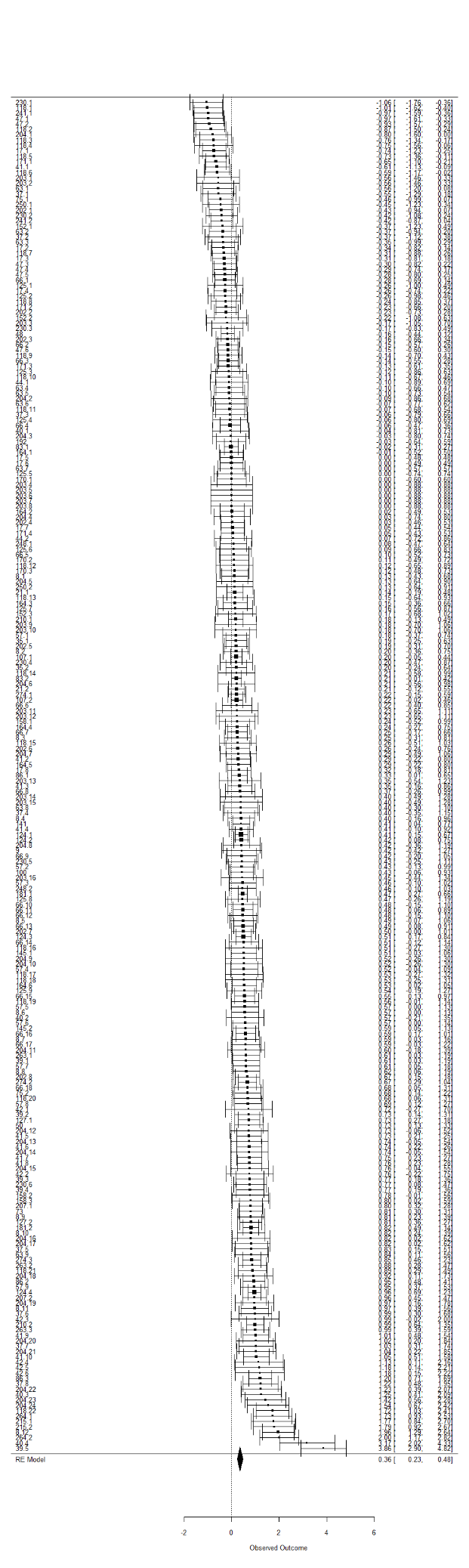
**

**Supplementary Figure 2.** Forest plot for Attention Studies.

**
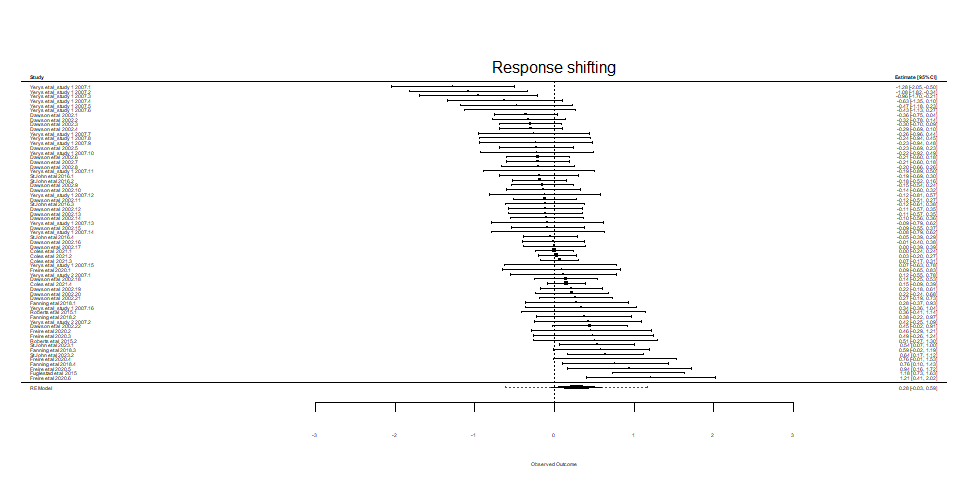
**

**
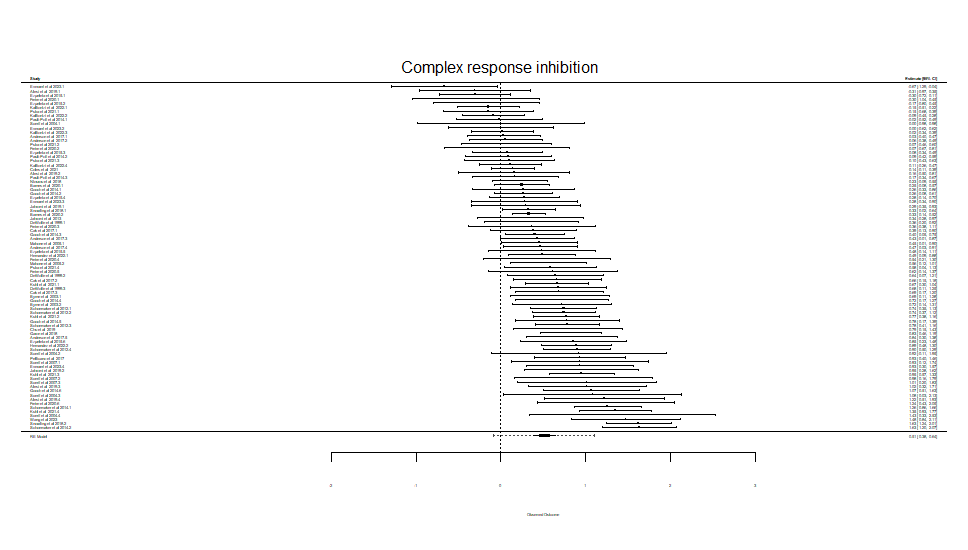
**

**
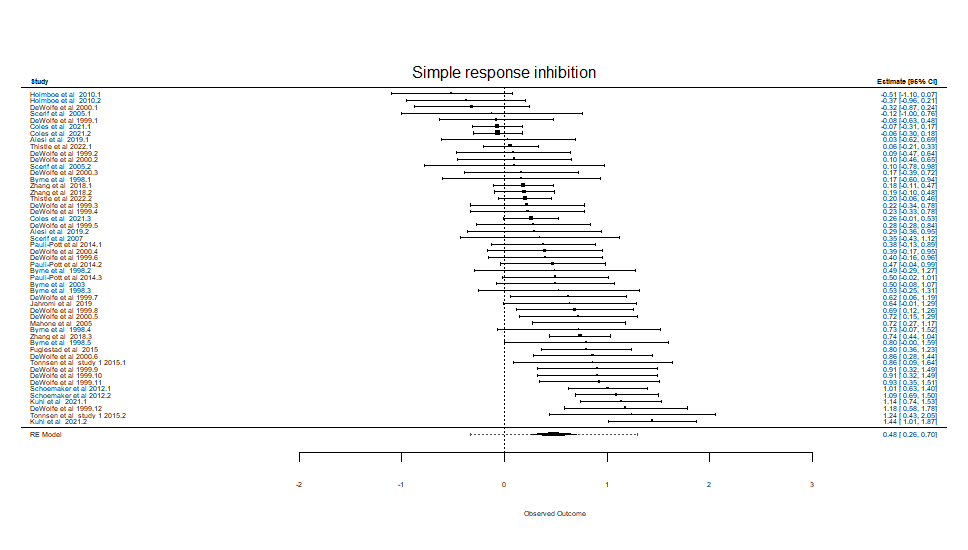
**

**
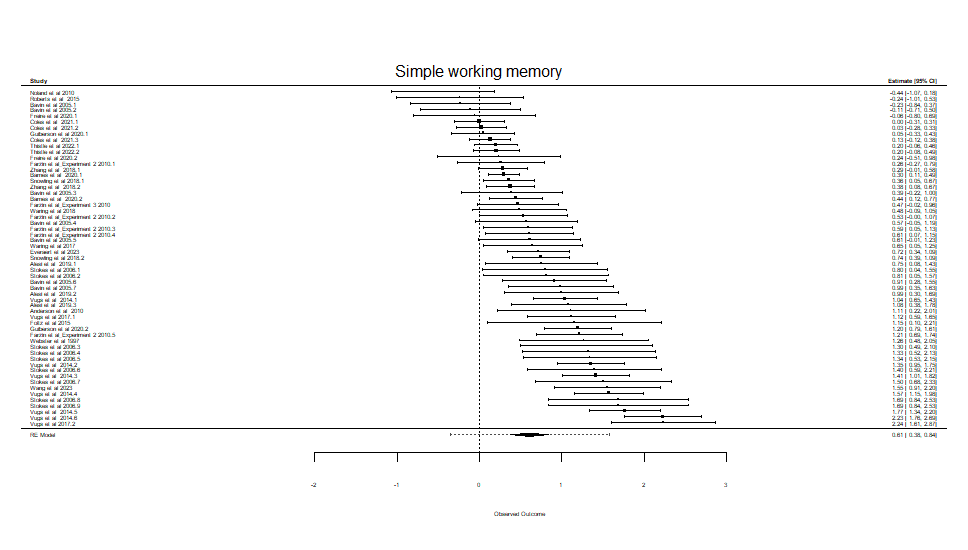
**

**
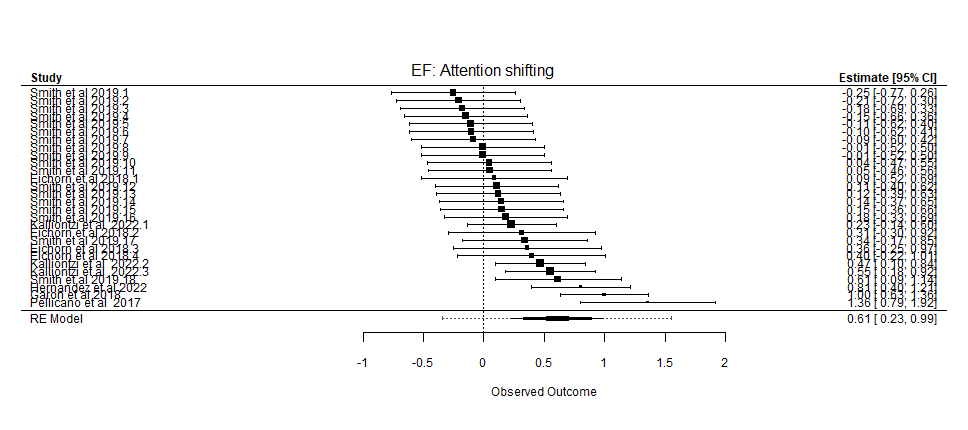
**

**
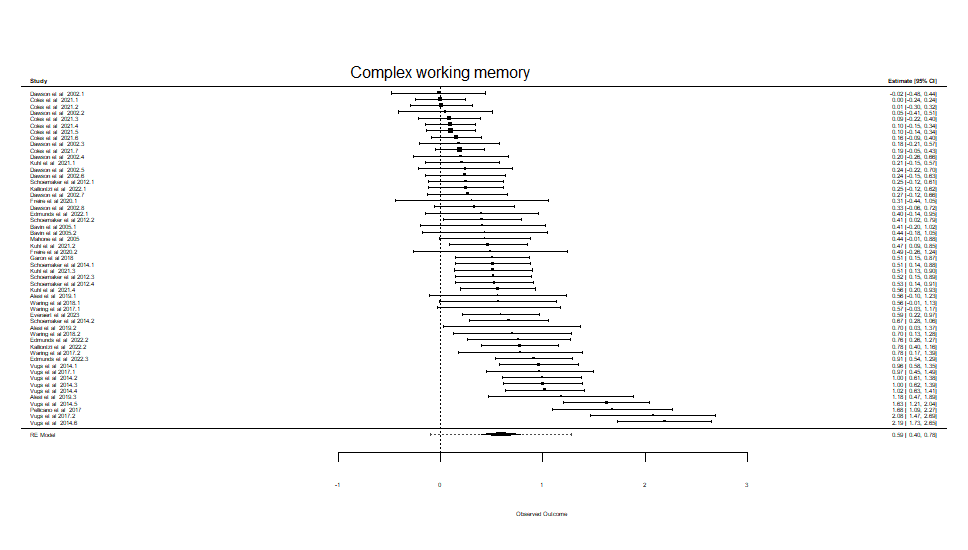
**

**
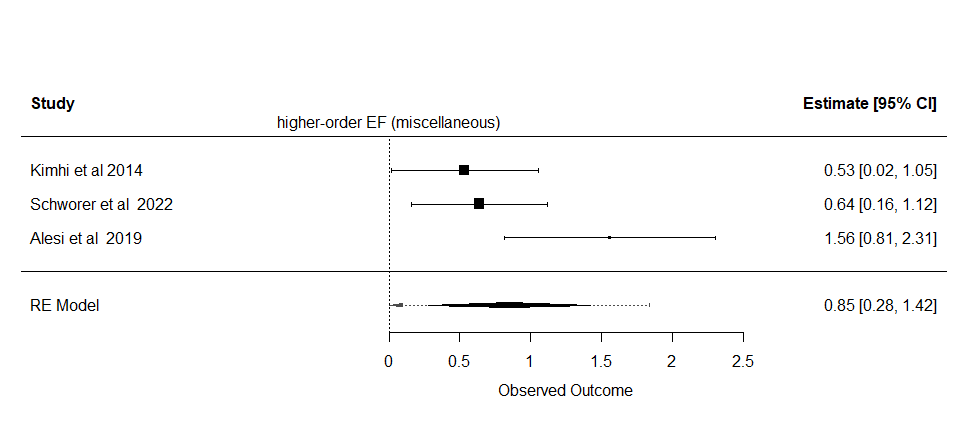
**

**
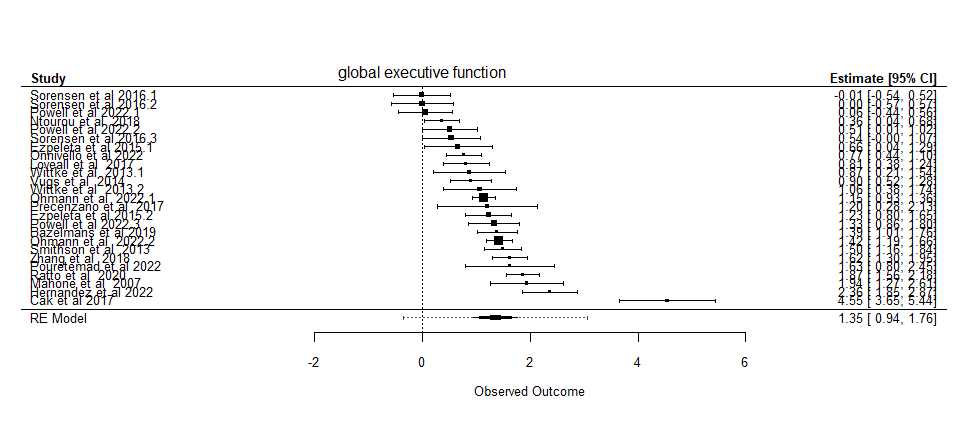
**

**Supplementary Figure 3.** Forest plots for Executive Function separated by domains.

**Supplementary Results 1.** Bayesian Model Meta-analysis.

The overall estimates from the Bayesian Model meta-analysis, which controlled for potential publication biases, showed an average Hedge’s g effect size of 0.600 (95% CI: 0.493-0.709) for executive functioning domains, with τ=0.468 (95%CI: 0.403-0.548) and rho = 0.653 (95% CI: 0.521-0.773). For attention, the effect size was d=0.348 (95% CI 0.225-0.473), with τ=0.482 (95% CI: 0.404-0.582) and rho = 0.580 (95% CI: 0.392-0.743). The Bayesian models used 2 out of 4 available models, with a prior probability of 0.5 and a posterior probability of 1.0, indicating strong evidence in favour of the effect for both EF (BF=5.10e+14) and attention (BF=2.43e+04). It also showed strong evidence of heterogeneity (EF: BF=6.59e+284; attention: BF=3.27e+71), and study-level differences (EF: BF=6.59e+284; attention: BF=3.27e+71).

**Supplementary Table 4.** Attention, Executive Function and Executive Function Components between Children with Neurodevelopmental Conditions compared to Neurotypical Children

| EF and Attention | | | |
| --- | --- | --- | --- |
| Measure | **Hedge’s g**  (CI 95%) | **p-value** | τ**^2^** |
| EF  (n = 64; k = 368) | 0.640  (0.525 – 0.755) | <.001*** | 0.192 |
| Attention  (n = 49; k = 251) | 0.357  (0.232 – 0.481) | <.001*** | 0.167 |

| EF domains: Performance-based Measures | | | |
| --- | --- | --- | --- |
| EF domain | **Hedge’s g**  (CI 95%) | **p-value** | τ**^2^** |
| Simple Working Memory  (n = 22; k = 62) | 0.788  (0.427 – 1.149) | <.001*** | 0.685 |
| Complex Working Memory  (n = 17; k = 58) | 0.655  (0.464 – 0.846) | <.001*** | 0.132 |
| Simple Response Inhibition  (n = 16; k = 51) | 0.481  (0.263 – 0.698) | <.001*** | 0.160 |
| Complex Response Inhibition  (n = 28; k = 94) | 0.618  (0.433 – 0.803) | <.001*** | 0.222 |
| Response Shifting  (n = 8; k = 63) | 0.280  (-0.033 – 0.593) | 0.080 | 0.182 |
| Attention Shifting  (n = 8; k = 33) | 1.00  (0.361 – 1.647) | 0.002** | 0.814 |
| Higher-Order EF  (n = 3; k = 3) | 0.852  (0.281 – 1.423) | 0.003* | 0.168 |

| EF components: Informant-based Measures | | | | |
| --- | --- | --- | --- | --- |
| EF subscales | **Hedge’s g**  (CI 95%) | **p-value** | | τ**^2^** |
| Inhibit  (n = 21; k = 29) | 1.181  (0.853 – 1.509) | <.0001*** | | 0.535 |
| Emotional Control  (n = 18; k = 24) | 0.853  (0.525 – 1.182) | <.0001*** | | 0.458 |
| Working Memory  (n = 18; k = 24) | 1.624  (1.281 – 1.967) | <.0001*** | | 0.497 |
| Plan/Organize  (n = 17; k = 22) | 1.277  (0.937 – 1.616) | <.0001*** | | 0.457 |
| Shift  (n = 17; k = 22) | 0.890  (0.529 – 1.251) | <.0001*** | | 0.523 |
| EF factors | **Hedge’s g**  (CI 95%) | | **p-value** | τ**^2^** |
| Inhibitory Self-Control  (n = 18; k = 27) | 1.061  (0.703 – 1.419) | <.0001*** | | 0.544 |
| Emergent Metacognition  (n = 17; k = 26) | 1.599  (1.195 – 2.00) | <.0001*** | | 0.661 |
| Flexibility  (n = 17; k = 26) | 0.974  (0.563 – 1.385) | <.0001*** | | 0.691 |

Abbreviations: EF, executive function; n, number of studies; k, number of outcomes; CI, confidence interval.

**Supplementary Table 5.** Moderator Analyses for Attention

| Attention  Categorical Moderator | | | |
| --- | --- | --- | --- |
| Moderator Variable  Test of Moderator | **Moderator grouping** | **Hedge’s g**  (CI 95%) | **p-value** |
| Age Group  QM (df = 2) = 16.67,  p < .001  τ^2^ = 0.13  (n = 49; k = 251) | Infancy  (n = 13; k = 56) | 0.051  (-0.165 – 0.267) | 0.644 |
|  | Toddlerhood  (n = 12; k = 64) | 0.275  (0.085 – 0.465) | 0.005** |
|  | Preschool  (n = 25; k = 131) | 0.548  (0.403 – 0.693) | <.001*** |
| Type of Measure  QM (df = 2) = 21.49,  p < .001  τ^2^ = 0.22  (n = 49, k = 251) | Performance-based  (n = 42; k = 230) | 0.265  (0.119 – 0.410) | <.001*** |
|  | Informant-based  (n = 9; k = 14) | 1.009  (0.699 – 1.320) | <.001*** |
|  | Physiological  (n = 3; k = 7) | 0.383  (-0.023 – 0.789) | 0.064 |
| NDC Diagnosis  QM (df = 10) = 34.60,  p < .001  τ^2^ = 0.17  (n = 49; k = 251) | IDD  (n = 3; k = 14) | 0.310  (0.067 – 0.552) | 0.012* |
|  | Communication Disorders  (n = 7; k = 22) | 0.747  (0.405 – 1.089) | <.001*** |
|  | Autism  (n = 17; k = 66) | 0.158  (-0.023 – 0.340) | 0.087 |
|  | ADHD  (n = 6; k = 39) | 0.643  (0.300 – 0.985) | <.001*** |
|  | Specific Learning Disorder  (n = 2; k = 3) | 0.488  (-0.108 – 1.083) | 0.108 |
|  | Genetic Disorder  (n = 8; k = 60) | 0.301  (0.051 – 0.550) | 0.018* |
|  | Cerebral Palsy  (n = 2; k = 3) | 0.273  (-0.422 – 0.968) | 0.441 |
|  | High-risk autism  (n = 11; k = 31) | 0.305  (0.098 – 0.513) | 0.004** |
|  | Comorbid ADHD + oppositional defiant disorder  (n = 1; k = 9) | 0.865  (0.449 – 1.281) | <.001*** |
|  | High-risk Communication Disorder  (n = 2; k = 2) | 0.016  (-0.411 – 0.444) | 0.940 |
|  | High-risk FASD  (n = 1; k = 2) | 0.208  (-0.606 – 1.023) | 0.616 |
| Continuous moderator | | | |
| Moderator Variable  Test of Moderator  (no. of studies) | **Moderator variable** | **β estimate** | **p-value** |
| Gender  QM (df = 1) = 0.03, p = 0.86  τ^2^ = 0.18  (n = 46; k = 239) | % of males in NDC group | <.001  (-.004 - .004) | 0.864 |

Abbreviations: n, number of studies; k = number of outcomes; CI, confidence interval; df, degrees of freedom; NDC, neurodevelopmental condition; IDD, intellectual disability disorder; ADHD, attention-deficit hyperactivity disorder; FASD, fetal alcohol spectrum disorder

**Supplementary Table 6.** Moderator Analyses for Executive Function

| EF  Categorical Moderator | | | |
| --- | --- | --- | --- |
| Moderator Variable  Test of Moderator | **Moderator grouping** | **Hedge’s g**  **(CI 95%)** | **p-value** |
| Age Group  QM (df = 2) = 12.18,  p = 0.002  τ^2^ = 0.17  (n = 64, k = 368) | Infancy  (n = 2; k = 3) | -0.444  (-1.122 – 0.234) | 0.199 |
|  | Toddlerhood  (n = 10; k = 64) | 0.482  (0.209 – 0.755) | <.001*** |
|  | Preschool  (n = 53; k = 301) | 0.698  (0.580 – 0.816) | <.001*** |
| Type of Measure  QM (df = 1) = 107.38,  p < .001  τ^2^ = 0.16  (n = 64, k = 368) | Performance-based  (n = 52; k = 339) | 0.482  (0.372 – 0.592) | <.001*** |
|  | Informant-based  (n = 20; k = 29) | 1.256  (1.098 – 1.414) | <.001*** |
|  | Physiological  (n = 0) | - |  |
| NDC Diagnosis  QM (df = 14) = 38.96,  p < .001  τ^2^ = 0.20  (n = 64, k = 368) | IDD  (n = 1; k = 1) | 0.233 (-0.233 – 0.698) | 0.3272 |
|  | Communication Disorders  (n = 15; k = 74) | 0.743 (0.497 – 0.989) | <.001*** |
|  | Autism  (n = 19; k = 76) | 0.712 (0.507 – 0.917) | <.001*** |
|  | ADHD  (n = 12; k = 70) | 0.663 (0.405 – 0.921) | <.001*** |
|  | Specific Learning Disorder  (n =2; k = 5) | 0.508 (-0.172 – 1.188) | 0.143 |
|  | Motor Disorders  (n = 1; = 13) | 0.675 (-0.214 – 1.564) | 0.137 |
|  | Genetic Disorders  (n = 9; k = 32) | 0.651 (0.356 – 0.946) | <.001*** |
|  | FASD  (n = 1; k = 2) | 0.982 (0.058 0 1.905) | 0.037* |
|  | Cerebral Palsy  (n = 2; k = 19) | 0.320 (-0.321 – 0.960) | 0.328 |
|  | High-risk Autism  (n = 4; k = 8) | 0.407 (0.080 – 0.734) | 0.015* |
|  | Mixed NDCs  (n = 3; k = 24) | 0.687 (0.446 – 0.928) | <.001*** |
|  | Comorbid ADHD + oppositional defiant disorder  (n = 3; k = 16) | 0.802 (0.514 – 1.090) | <.001*** |
|  | High-risk ADHD  (n = 1; k = 6) | 0.262 (-0.631 – 1.155) | 0.566 |
|  | High-risk Communication Disorders  (n = 2; k = 4) | 0.148 (-0.184 – 0.481) | 0.382 |
|  | High-risk FASD  (n = 1; k = 18) | 0.074  (-0.796 – 0.945) | 0.867 |
| Continuous moderator | | | |
| Moderator Variable  Test of Moderator  (no. of studies) | **Moderator variable** | **β estimate** | **p-value** |
| Gender  QM (df = 1) = 0.74  τ^2^ = 0.19  (n = 61, k = 348) | % of males in NDC group | 0.001  (-0.002 – 0.004) | 0.389 |

Abbreviations: EF, executive function; CI, confidence interval; df, degrees of freedom; n, number of studies; k, number of outcomes; NDC, neurodevelopmental condition; IDD, intellectual disability disorder; ADHD, attention-deficit hyperactivity disorder; FASD, fetal alcohol spectrum disorder

**Supplementary Table 7.** Individual Studies in the Qualitative Analysis

| **Author & year** | **Diagnostic Group** | **Technique** | **Task** | **Major finding** | **Control sample size** | **Mean age (years)** | **Gender distribution (% of Males in NDC group)** | **Quality Assessment** |
| --- | --- | --- | --- | --- | --- | --- | --- | --- |
| Zielinski et al 2022(124) | Autism  (n = 122) | Structural MRI | NA | The ECN was observed to be spatially confined in autistic children, showing limited structural covariance outside of the dorsolateral PFC, while there was extensive structural covariance across the dorsolateral PFC, ventromedial PFC, and the ACC in TD children | 122 | 3.13 | 50 | Good |
| Zhang et al 2020(125) | ADHD  (n = 25) | ERP using EEG | Three-stimulus oddball paradigm | Decreased mismatch negativity and amplitude of the P3a at the Cz site (EEG) in children with ADHD, suggesting evidence of difficulties in preattentive change detection and attention shifting, respectively. No significant differences were found at the Fz site. | 22 | 4.84 | 40 | Good |
| Hou et al 2022(126) | Autism  (n = 45) | fNIRS | Attention task | Autistic children showed significantly lower activation in the right dorsolateral PFC compared to TD children while watching a robot video. Autistic children showed a significantly lower activation in the left dorsolateral PFC compared to TD children while watching a baseline video. No significant differences were found in the left and right dorsolateral PFC while children watched the robot and baseline videos, respectively. | 53 | 4.89 | 91.11 | Good |
| Goodwin et al 2021(127) | High-risk ADHD (n = 27), high-risk Autism (n = 77), high-risk ADHD and Autism (n = 20) | EEG | Attention task | No significant differences in frontal activity between children at risk for ADHD and/or Autism and TD children while watching a non-social video | 27 | 0.89, 0.88, 0.88 | 55.56, 50.65, 60 | Good |
| Baving et al 1999(128) | ADHD (n = 20), ADHD (n = 8) | EEG | NA | Compromised resting neural activity in the frontal regions in children with ADHD, but with gender-specific effects. Boys with ADHD showed a reduced right frontal activity compared to TD boys, while girls with ADHD showed reduced left frontal activity compared to TD girls | 15, 23 | 4.5, 4.5 | 100, 0 | Good |

Abbreviations: NA, not applicable; ECN, executive control network, PFC, prefrontal cortex; ACC, anterior cingulate cortex; TD, typically developing; ADHD, attention-deficit hyperactivity disorder; ERP, event-related potential; EEG, electroencephalogram; Cz, midline central; Fz, midline frontal; fNIRs, functional near-infrared spectroscopy.

**Supplementary Results 2.** Qualitative Analysis

One study used structural covariance magnetic resonance imaging (MRI) to compare the executive control network (ECN) between autistic preschool children and TD peers, which revealed that the ECN (centred around the dorsolateral PFC [dlPFC]) in autistic children is reduced both in volume and extent(124) (see Figure 3). Another study used functional near-infrared spectroscopy (fNIRS) to demonstrate that autistic preschool children show a significantly lower activation in the dlPFC compared to TD peers, while watching non-social video.(126) Two studies using electroencephalogram (EEG) found that preschool children with ADHD show a compromised resting frontal activity(128) and atypical event-related potentials (ERPs) at the Cz site (EEG) while completing an oddball paradigm.(125)

**References**

1. Bishop DV. Which neurodevelopmental disorders get researched and why? PLoS One. 2010;5(11):e15112.

2. Garon N, Bryson SE, Smith IM. Executive function in preschoolers: A review using an integrative framework. Psychol Bull. 2008;134(1):31-60.

3. Gioia GA, Andrwes K, Isquith PK. Behavior rating inventory of executive function-preschool version (BRIEF-P): Psychological Assessment Resources Odessa, FL; 1996.

4. Putnam SP, Ellis LK, Rothbart MK. The structure of temperament from infancy through adolescence. Advances in research on temperament. 2001;165:182.

5. Anderson PJ, Reidy N. Assessing executive function in preschoolers. Neuropsychol Rev. 2012;22(4):345-60.

6. Hendry A, Jones EJH, Charman T. Executive function in the first three years of life: Precursors, predictors and patterns. Developmental Review. 2016;42:1-33.

7. Keenan K, Shaw D. Developmental and social influences on young girls' early problem behavior. Psychol Bull. 1997;121(1):95-113.

8. Alesi M, Pecoraro D, Pepi A. Executive functions in kindergarten children at risk for developmental coordination disorder. European Journal of Special Needs Education. 2018;34(3):285-96.

9. Aljahlan Y, Spaulding TJ. The Impact of Manipulating Attentional Shifting Demands on Preschool Children With Specific Language Impairment. J Speech Lang Hear Res. 2019;62(2):324-36.

10. Anderson JD, Wagovich SA. Relationships among linguistic processing speed, phonological working memory, and attention in children who stutter. J Fluency Disord. 2010;35(3):216-34.

11. Anderson JD, Wagovich SA. Explicit and Implicit Verbal Response Inhibition in Preschool-Age Children Who Stutter. J Speech Lang Hear Res. 2017;60(4):836-52.

12. Smith H, Blaser E, Carter AS, Kaldy Z. Resistance to distraction during visual search in 2-year-old toddlers with and without Autism Spectrum Disorder: An eye-tracking study. unpublished.

13. Bavin EL, Wilson PH, Maruff P, Sleeman F. Spatio-visual memory of children with specific language impairment: evidence for generalized processing problems. Int J Lang Commun Disord. 2005;40(3):319-32.

14. Bazelmans T, Jones EJH, Ghods S, Corrigan S, Toth K, Charman T, Webb SJ. Heart rate mean and variability as a biomarker for phenotypic variation in preschoolers with autism spectrum disorder. Autism Res. 2019;12(1):39-52.

15. Bradshaw J, Gillespie S, Klaiman C, Klin A, Saulnier C. Early emergence of discrepancy in adaptive behavior and cognitive skills in toddlers with autism spectrum disorder. Autism. 2019;23(6):1485-96.

16. Brown JH, Johnson MH, Paterson SJ, Gilmore R, Longhi E, Karmiloff-Smith A. Spatial representation and attention in toddlers with Williams syndrome and Down syndrome. Neuropsychologia. 2003;41(8):1037-46.

17. Bryson S, Garon N, McMullen T, Brian J, Zwaigenbaum L, Armstrong V, et al. Impaired disengagement of attention and its relationship to emotional distress in infants at high-risk for autism spectrum disorder. J Clin Exp Neuropsychol. 2018;40(5):487-501.

18. Byrne JM, Bawden HN, Beattie T, DeWolfe NA. Risk for injury in preschoolers: relationship to attention deficit hyperactivity disorder. Child Neuropsychol. 2003;9(2):142-51.

19. Byrne JM, DeWolfe NA, Bawden HN. Assessment of attention-deficit hyperactivity disorder in preschoolers. Child Neuropsychology. 1998;4(1):49-66.

20. Cak HT, Cengel Kultur SE, Gokler B, Oktem F, Taskiran C. The Behavior Rating Inventory of Executive Function and Continuous Performance Test in Preschoolers with Attention Deficit Hyperactivity Disorder. Psychiatry Investig. 2017;14(3):260-70.

21. Cardon T, Azuma T. Visual attending preferences in children with autism spectrum disorders: A comparison between live and video presentation modes. Research in Autism Spectrum Disorders. 2012;6(3):1061-7.

22. Chawarska K, Klin A, Volkmar F. Automatic attention cueing through eye movement in 2-year-old children with autism. Child Dev. 2003;74(4):1108-22.

23. Cheung CHM, Bedford R, Johnson MH, Charman T, Gliga T, team B. Visual search performance in infants associates with later ASD diagnosis. Dev Cogn Neurosci. 2018;29:4-10.

24. Clark CE, Conture EG, Walden TA, Lambert WE. Speech-Language Dissociations, Distractibility, and Childhood Stuttering. Am J Speech Lang Pathol. 2015;24(3):480-503.

25. Constantinou JC, Adamson-Macedo EN, Mirmiran M, Fleisher BE. Movement, imaging and neurobehavioral assessment as predictors of cerebral palsy in preterm infants. J Perinatol. 2007;27(4):225-9.

26. Dawson G, Munson J, Estes A, Osterling J, McPartland J, Toth K, et al. Neurocognitive function and joint attention ability in young children with autism spectrum disorder versus developmental delay. Child Dev. 2002;73(2):345-58.

27. DeWolfe NA, Byrne JM, Bawden HN. Early clinical assessment of attention. Clin Neuropsychol. 1999;13(4):458-73.

28. DeWolfe NA, Byrne JM, Bawden HN. Preschool inattention and impulsivity-hyperactivity: Development of a clinic-based assessment protocol. Journal of attention disorders. 2000;4(2):80-90.

29. Edmunds SR, Colman C, Vidal P, Faja S. Brief Report: Examining the Links Between Language Processes and Working Memory Impairments in Toddlers and Preschoolers with ASD. J Autism Dev Disord. 2022;52(4):1872-80.

30. Eichorn N, Marton K, Pirutinsky S. Cognitive flexibility in preschool children with and without stuttering disorders. Journal of Fluency Disorders. 2018;57:37-50.

31. Elsabbagh M, Volein A, Holmboe K, Tucker L, Csibra G, Baron-Cohen S, et al. Visual orienting in the early broader autism phenotype: disengagement and facilitation. J Child Psychol Psychiatry. 2009;50(5):637-42.

32. Elsabbagh M, Fernandes J, Webb SJ, Dawson G, Charman T, Johnson MH, British Autism Study of Infant Siblings T. Disengagement of visual attention in infancy is associated with emerging autism in toddlerhood. Biol Psychiatry. 2013;74(3):189-94.

33. Ezpeleta L, Granero R. Executive functions in preschoolers with adhd, odd, and comorbid adhd-odd: Evidence from ecological and performance-based measures. Journal of Neuropsychology. 2015;9(2):258-70.

34. Fanning PAJ, Hocking DR, Dissanayake C, Vivanti G. Delineation of a spatial working memory profile using a non-verbal eye-tracking paradigm in young children with autism and Williams syndrome. Child Neuropsychol. 2018;24(4):469-89.

35. Farzin F, Rivera SM. Dynamic Object Representations in Infants with and without Fragile X Syndrome. Frontiers in human neuroscience. 2010;4:12-.

36. Ference J, Curtin S. Attention to lexical stress and early vocabulary growth in 5-month-olds at risk for autism spectrum disorder. J Exp Child Psychol. 2013;116(4):891-903.

37. Waring R, Eadie P, Rickard Liow S, Dodd B. The phonological memory profile of preschool children who make atypical speech sound errors. Clin Linguist Phon. 2018;32(1):28-45.

38. Foltz A, Thiele K, Kahsnitz D, Stenneken P. Children's syntactic-priming magnitude: lexical factors and participant characteristics. J Child Lang. 2015;42(4):932-45.

39. Freire TC, Osorio AAC. Executive functions and drawing in young children with cerebral palsy: Comparisons with typical development. Child Neuropsychol. 2020;26(5):635-48.

40. Fuglestad AJ, Whitley ML, Carlson SM, Boys CJ, Eckerle JK, Fink BA, Wozniak JR. Executive functioning deficits in preschool children with Fetal Alcohol Spectrum Disorders. Child Neuropsychology. 2015;21(6):716-31.

41. Garon N, Smith IM, Bryson SE. Early executive dysfunction in ASD: Simple versus complex skills. Autism Research. 2018;11(2):318-30.

42. Gooch D, Hulme C, Nash HM, Snowling MJ. Comorbidities in preschool children at family risk of dyslexia. J Child Psychol Psychiatry. 2014;55(3):237-46.

43. Guiberson MM, Rodriguez BL. Working Memory and Linguistic Performance of Dual Language Learners With and Without Developmental Language Disorders. Am J Speech Lang Pathol. 2020;29(3):1301-6.

44. Holmboe K, Elsabbagh M, Volein A, Tucker LA, Baron-Cohen S, Bolton P, et al. Frontal cortex functioning in the infant broader autism phenotype. Infant Behav Dev. 2010;33(4):482-91.

45. Jahromi LB, Bryce CI, Swanson J. The importance of self-regulation for the school and peer engagement of children with high-functioning autism. Research in Autism Spectrum Disorders. 2013;7(2):235-46.

46. Jahromi LB, Chen Y, Dakopolos AJ, Chorneau A. Delay of gratification in preschoolers with and without autism spectrum disorder: Individual differences and links to executive function, emotion regulation, and joint attention. Autism. 2019;23(7):1720-31.

47. St John T, Estes AM, Dager SR, Kostopoulos P, Wolff JJ, Pandey J, et al. Emerging Executive Functioning and Motor Development in Infants at High and Low Risk for Autism Spectrum Disorder. Frontiers in Psychology. 2016;7.

48. Joyce A, Elphick H, Farquhar M, Gringras P, Evans H, Bucks RS, et al. Obstructive Sleep Apnoea Contributes to Executive Function Impairment in Young Children with Down Syndrome. Behavioral sleep medicine. 2020;18(5):611-21.

49. Coles CD, Kable JA, Granovska IV, Pashtepa AO, Wertelecki W, Chambers CD, Cifasd. Measurement of neurodevelopmental effects of prenatal alcohol exposure in Ukrainian preschool children. Child Neuropsychol. 2021;27(8):1088-103.

50. Kalliontzi E, Ralli AM, Palikara O, Roussos P. Examining the relationship between oral language skills and executive functions: Evidence from Greek-speaking 4-5-year-old children with and without Developmental Language Disorder. Res Dev Disabil. 2022;124:104215.

51. Kapa LL, Erikson JA. The Relationship Between Word Learning and Executive Function in Preschoolers With and Without Developmental Language Disorder. J Speech Lang Hear R. 2020;63(7):2293-307.

52. Kapa LL, Plante E, Doubleday K. Applying an Integrative Framework of Executive Function to Preschoolers With Specific Language Impairment. J Speech Lang Hear R. 2017;60(8):2170-84.

53. Kimhi Y, Shoam-Kugelmas D, Ben-Artzi GA, Ben-Moshe I, Bauminger-Zviely N. Theory of Mind and Executive Function in Preschoolers with Typical Development Versus Intellectually Able Preschoolers with Autism Spectrum Disorder. Journal of Autism and Developmental Disorders. 2014;44(9):2341-54.

54. Krakow JB, Kopp CB. Sustained attention in young Down syndrome children. Topics in Early Childhood Special Education. 1982;2(2):32-42.

55. Krakow JB, Kopp CB. The effects of developmental delay on sustained attention in young children. Child Development. 1983:1143-55.

56. Kuhl E, Geeraerts SB, Dekovic M, Schoemaker K, Bunte T, Espy KA, Matthys W. Trajectories of Executive Functions and ADHD Symptoms in Preschoolers and the Role of Negative Parental Discipline. Dev Neuropsychol. 2021;46(8):555-73.

57. Loveall SJ, Conners FA, Tungate AS, Hahn LJ, Osso TD. A cross-sectional analysis of executive function in Down syndrome from 2 to 35 years. J Intellect Disabil Res. 2017;61(9):877-87.

58. Macari SL, Koller J, Campbell DJ, Chawarska K. Temperamental markers in toddlers with autism spectrum disorder. J Child Psychol Psychiatry. 2017;58(7):819-28.

59. Maestro S, Muratori F, Cavallaro MC, Pei F, Stern D, Golse B, Palacio-Espasa F. Attentional skills during the first 6 months of age in autism spectrum disorder. J Am Acad Child Adolesc Psychiatry. 2002;41(10):1239-45.

60. Maestro S, Muratori F, Cavallaro MC, Pecini C, Cesari A, Paziente A, et al. How young children treat objects and people: an empirical study of the first year of life in autism. Child psychiatry and human development. 2005;35:383-96.

61. Mahone EM, Hoffman J. Behavior ratings of executive function among preschoolers with ADHD. The Clinical Neuropsychologist. 2007;21(4):569-86.

62. Mahone EM, Pillion JP, Hoffman J, Hiemenz JR, Denckla MB. Construct validity of the auditory continuous performance test for preschoolers. Dev Neuropsychol. 2005;27(1):11-33.

63. Ntourou K, Anderson JD, Wagovich SA. Executive function and childhood stuttering: Parent ratings and evidence from a behavioral task. J Fluency Disord. 2018;56:18-32.

64. Parhiala P, Torppa M, Eklund K, Aro T, Poikkeus AM, Heikkilä R, Ahonen T. Psychosocial functioning of children with and without dyslexia: A follow‐up study from ages four to nine. Dyslexia. 2015;21(3):197-211.

65. Pellicano E, Kenny L, Brede J, Klaric E, Lichwa H, McMillin R. Executive function predicts school readiness in autistic and typical preschool children. Cognitive Development. 2017;43:1-13.

66. Powell K, Macari S, Brennan-Wydra E, Feiner H, Butler M, Goncalves Fortes D, et al. Elevated symptoms of executive dysfunction predict lower adaptive functioning in 3-year-olds with autism spectrum disorder. Autism Res. 2022;15(7):1336-47.

67. Putra PU, Shima K, Alvarez SA, Shimatani K. Identifying autism spectrum disorder symptoms using response and gaze behavior during the Go/NoGo game CatChicken. Sci Rep. 2021;11(1):22012.

68. Ratto AB, Potvin D, Pallathra AA, Saldana L, Kenworthy L. Parents report fewer executive functioning problems and repetitive behaviors in young dual-language speakers with autism. Child Neuropsychology. 2020;26(7):917-33.

69. Roberts LV, Richmond JL. Preschoolers with Down syndrome do not yet show the learning and memory impairments seen in adults with Down syndrome. Dev Sci. 2015;18(3):404-19.

70. Sasson NJ, Elison JT, Turner-Brown LM, Dichter GS, Bodfish JW. Brief report: Circumscribed attention in young children with autism. J Autism Dev Disord. 2011;41(2):242-7.

71. Wittke K, Spaulding TJ, Schechtman CJ. Specific language impairment and executive functioning: parent and teacher ratings of behavior. Am J Speech Lang Pathol. 2013;22(2):161-72.

72. Schneider H, Ryan M, Mahone EM. Parent versus teacher ratings on the BRIEF-preschool version in children with and without ADHD. Child neuropsychology. 2020;26(1):113-28.

73. Schoemaker K, Bunte T, Espy KA, Dekovic M, Matthys W. Executive functions in preschool children with ADHD and DBD: an 18-month longitudinal study. Dev Neuropsychol. 2014;39(4):302-15.

74. Schwenk KA, Conture EG, Walden TA. Reaction to background stimulation of preschool children who do and do not stutter. J Commun Disord. 2007;40(2):129-41.

75. Skogan AH, Zeiner P, Egeland J, Urnes AG, Reichborn-Kjennerud T, Aase H. Parent ratings of executive function in young preschool children with symptoms of attention-deficit/-hyperactivity disorder. Behav Brain Funct. 2015;11:16.

76. Smith H, Carter AS, Blaser E, Kaldy Z. Successful attentional set-shifting in 2-year-olds with and without Autism Spectrum Disorder. PLoS One. 2019;14(3):e0213903.

77. Waring R, Eadie P, Rickard Liow S, Dodd B. Do children with phonological delay have phonological short-term and phonological working memory deficits? Child Language Teaching and Therapy. 2016;33(1):33-46.

78. Tonnsen BL, Richards JE, Roberts JE. Heart rate-defined sustained attention in infants at risk for autism. J Neurodev Disord. 2018;10(1):7.

79. Wang Q, Chang J, Chawarska K. Atypical Value-Driven Selective Attention in Young Children With Autism Spectrum Disorder. JAMA Netw Open. 2020;3(5):e204928.

80. Vugs B, Hendriks M, Cuperus J, Verhoeven L. Working memory performance and executive function behaviors in young children with SLI. Res Dev Disabil. 2014;35(1):62-74.

81. Barnes MA, Clemens NH, Fall A-M, Roberts G, Klein A, Starkey P, et al. Cognitive predictors of difficulties in math and reading in pre-kindergarten children at high risk for learning disabilities. Journal of Educational Psychology. 2020;112(4):685-700.

82. Hernandez ML, Spiegel JA, Coxe S, Dick AS, Graziano PA. Individual Differences in Germ Spreading Behaviors Among Children With Attention-Deficit/Hyperactivity Disorder: The Role of Executive Functioning. Journal of pediatric psychology. 2022;47(8):892-904.

83. Noland JS, Steven Reznick J, Stone WL, Walden T, Sheridan EH. Better working memory for non-social targets in infant siblings of children with Autism Spectrum Disorder. Dev Sci. 2010;13(1):244-51.

84. Pauli-Pott U, Roller A, Heinzel-Gutenbrunner M, Mingebach T, Dalir S, Becker K. Inhibitory control and delay aversion in unaffected preschoolers with a positive family history of attention deficit hyperactivity disorder. Journal of Child Psychology and Psychiatry. 2014;55(10):1117-24.

85. Ruskin EM, Kasari C, Mundy P, Sigman M. Attention to people and toys during social and object mastery in children with Down syndrome. American journal of mental retardation: AJMR. 1994;99(1):103-11.

86. Sacrey LA, Bryson SE, Zwaigenbaum L. Prospective examination of visual attention during play in infants at high-risk for autism spectrum disorder: a longitudinal study from 6 to 36 months of age. Behav Brain Res. 2013;256:441-50.

87. Scerif G, Cornish K, Wilding J, Driver J, Karmiloff-Smith A. Visual search in typically developing toddlers and toddlers with Fragile X or Williams syndrome. Developmental science. 2004;7(1):116-30.

88. Scerif G, Cornish K, Wilding J, Driver J, Karmiloff-Smith A. Delineation of early attentional control difficulties in fragile X syndrome: focus on neurocomputational changes. Neuropsychologia. 2007;45(8):1889-98.

89. Schoemaker K, Bunte T, Wiebe SA, Espy KA, Dekovic M, Matthys W. Executive function deficits in preschool children with ADHD and DBD. J Child Psychol Psychiatry. 2012;53(2):111-9.

90. Schworer EK, Fidler DJ, Kaur M, Needham AW, Prince MA, Daunhauer LA. Infant precursors of executive function in Down syndrome. J Intellect Disabil Res. 2022;66(1-2):108-20.

91. Skogan AH, Zeiner P, Egeland J, Rohrer-Baumgartner N, Urnes AG, Reichborn-Kjennerud T, Aase H. Inhibition and working memory in young preschool children with symptoms of ADHD and/or oppositional-defiant disorder. Child Neuropsychol. 2014;20(5):607-24.

92. Snowling MJ, Gooch D, McArthur G, Hulme C. Language Skills, but Not Frequency Discrimination, Predict Reading Skills in Children at Risk of Dyslexia. Psychol Sci. 2018;29(8):1270-82.

93. Sorensen K, Liverod JR, Lerdal B, Vestrheim IE, Skranes J. Executive functions in preschool children with cerebral palsy--Assessment and early intervention--A pilot study. Dev Neurorehabil. 2016;19(2):111-6.

94. Tonnsen BL, Grefer ML, Hatton DD, Roberts JE. Developmental trajectories of attentional control in preschool males with fragile X syndrome. Res Dev Disabil. 2015;36C:62-71.

95. Yerys BE, Hepburn SL, Pennington BF, Rogers SJ. Executive function in preschoolers with autism: evidence consistent with a secondary deficit. J Autism Dev Disord. 2007;37(6):1068-79.

96. Onnivello S, Colaianni S, Pulina F, Locatelli C, Marcolin C, Ramacieri G, et al. Executive functions and adaptive behaviour in individuals with Down syndrome. J Intellect Disabil Res. 2022;66(1-2):32-49.

97. Precenzano F, Ruberto M, Parisi L, Salerno M, Maltese A, Vagliano C, et al. EXECUTIVE FUNCTIONING IN PRESCHOOL CHILDREN AFFECTED BY AUTISM SPECTRUM DISOR-DER: A PILOT STUDY. Acta Medica. 2017;33:35.

98. Smithson PE, Kenworthy L, Wills MC, Jarrett M, Atmore K, Yerys BE. Real world executive control impairments in preschoolers with autism spectrum disorders. J Autism Dev Disord. 2013;43(8):1967-75.

99. Watson LR, Roberts JE, Baranek GT, Mandulak KC, Dalton JC. Behavioral and physiological responses to child-directed speech of children with autism spectrum disorders or typical development. J Autism Dev Disord. 2012;42(8):1616-29.

100. Wang L, Liang X, Jiang B, Wu Q, Jiang L. What ability can predict mathematics performance in typically developing preschoolers and those with autism spectrum disorder? Journal of Autism and Developmental Disorders. 2023;53(5):2062-77.

101. McClain MB, Golson ME, Murphy LE. Executive functioning skills in early childhood children with autism, intellectual disability, and co-occurring autism and intellectual disability. Res Dev Disabil. 2022;122:104169.

102. Rudling M, Nystrom P, Bolte S, Falck-Ytter T. Larger pupil dilation to nonsocial sounds in infants with subsequent autism diagnosis. J Child Psychol Psychiatry. 2022;63(7):793-801.

103. Scerif G, Karmiloff-Smith A, Campos R, Elsabbagh M, Driver J, Cornish K. To look or not to look? Typical and atypical development of oculomotor control. J Cogn Neurosci. 2005;17(4):591-604.

104. Zhang HF, Shuai L, Zhang JS, Wang YF, Lu TF, Tan X, et al. Neuropsychological Profile Related with Executive Function of Chinese Preschoolers with Attention-Deficit/Hyperactivity Disorder: Neuropsychological Measures and Behavior Rating Scale of Executive Function-Preschool Version. Chin Med J (Engl). 2018;131(6):648-56.

105. Zwaigenbaum L, Bryson S, Rogers T, Roberts W, Brian J, Szatmari P. Behavioral manifestations of autism in the first year of life. Int J Dev Neurosci. 2005;23(2-3):143-52.

106. Ohmann S, Wurzer M, Popow C. Attention-deficit hyperactivity disorder and executive dysfunction in preschool children. A comparison of NEPSY and BRIEF-P assessments. Encephale. 2022;48(3):232-40.

107. Berger SE, Harbourne RT, Guallpa Lliguichuzhca CL. Sit Still and Pay Attention! Trunk Movement and Attentional Resources in Infants with Typical and Delayed Development. Phys Occup Ther Pediatr. 2019;39(1):48-59.

108. Chu FW, vanMarle K, Hoard MK, Nugent L, Scofield JE, Geary DC. Preschool deficits in cardinal knowledge and executive function contribute to longer-term mathematical learning disability. J Exp Child Psychol. 2019;188:104668.

109. St John T, Estes AM, Hazlett HC, Marrus N, Burrows CA, Donovan K, et al. Association of Sex With Neurobehavioral Markers of Executive Function in 2-Year-Olds at High and Low Likelihood of Autism. JAMA network open. 2023;6(5):e2311543-e.

110. Vugs B, Hendriks M, Cuperus J, Knoors H, Verhoeven L. Developmental Associations Between Working Memory and Language in Children With Specific Language Impairment: A Longitudinal Study. J Speech Lang Hear Res. 2017;60(11):3284-94.

111. Fitch A, Valadez A, Ganea PA, Carter AS, Kaldy Z. Toddlers with Autism Spectrum Disorder Can Use Language to Update Their Expectations About the World. Journal of autism and developmental disorders. 2019;49(2):429-40.

112. Afshar M, Zarifian T, Khorrami Banaraki A, Noroozi M. Executive functions in Persian-speaking preschool children with speech sound disorders and comparison with their typically developing peers. Applied neuropsychology Child. 2022;11(4):702-12.

113. Everaert E, Vorstman JAS, Selten IS, Slieker MG, Wijnen F, Boerma TD, Houben ML. Executive functioning in preschoolers with 22q11.2 deletion syndrome and the impact of congenital heart defects. J Neurodev Disord. 2023;15(1):15.

114. Dimachkie Nunnally A, Baczewski L, Sterrett K, Holbrook A, Kaiser A, Kasari C. Profiles and trajectories of executive functioning in young children with Down syndrome. J Intellect Disabil Res. 2023;67(3):254-70.

115. Thistle JE, Ramos A, Roell KR, Choi G, Manley CK, Hall AM, et al. Prenatal organophosphorus pesticide exposure and executive function in preschool-aged children in the Norwegian Mother, Father and Child Cohort Study (MoBa). Environ Res. 2022;212(Pt D):113555.

116. Pouretemad HR, Sadeghi S, Badv RS, Brand S. Differentiating Post-Digital Nannying Autism Syndrome from Autism Spectrum Disorders in Young Children: A Comparative Cross-Sectional Study. J Clin Med. 2022;11(22).

117. Webster PE, Plante AS, Couvillion LM. Phonologic Impairment and Prereading: Update on a Longitudinal Study. Journal of learning disabilities. 1997;30(4):365-75.

118. Stokes SF, Wong AMY, Fletcher P, Leonard LB. Nonword Repetition and Sentence Repetition as Clinical Markers of Specific Language Impairment: The Case of Cantonese. Journal of speech, language, and hearing research. 2006;49(2):219-36.

119. Afshar MR, Ghorbani A, Rashedi V, Jalilevand N, Kamali M. Working memory span in Persian-speaking children with speech sound disorders and normal speech development. Int J Pediatr Otorhinolaryngol. 2017;101:117-22.

120. McClain MB, Golson ME, Murphy LE. Executive functioning skills in early childhood children with autism, intellectual disability, and co-occurring autism and intellectual disability. Research in developmental disabilities. 2022;122:104169.

121. Kapa LL, Plante E, Doubleday K. Applying an integrative framework of executive function to preschoolers with specific language impairment. Journal of Speech, Language, and Hearing Research. 2017;60(8):2170-84.

122. Schoemaker K, Bunte T, Wiebe SA, Espy KA, Dekovic M, Matthys W. Executive function deficits in preschool children with ADHD and DBD. J Child Psychol Psychiatry. 2012;53(2):111-9.

123. Kuhl E, Geeraerts SB, Dekovic M, Schoemaker K, Bunte T, Espy KA, Matthys W. Trajectories of Executive Functions and ADHD Symptoms in Preschoolers and the Role of Negative Parental Discipline. Developmental Neuropsychology. 2021;46(8):555-73.

124. Zielinski BA, Andrews DS, Lee JK, Solomon M, Rogers SJ, Heath B, et al. Sex-dependent structure of socioemotional salience, executive control, and default mode networks in preschool-aged children with autism. Neuroimage. 2022;257:119252.

125. Zhang J, Qiu M, Pan J, Zhao L. The preattentive change detection in preschool children with attention deficit hyperactivity disorder: a mismatch negativity study. Neuroreport. 2020;31(10):776-9.

126. Hou S, Liu N, Zou J, Yin X, Liu X, Zhang S, et al. Young children with autism show atypical prefrontal cortical responses to humanoid robots: An fNIRS study. Int J Psychophysiol. 2022;181:23-32.

127. Goodwin A, Hendry A, Mason L, Bazelmans T, Begum Ali J, Pasco G, et al. Behavioural Measures of Infant Activity but Not Attention Associate with Later Preschool ADHD Traits. Brain Sci. 2021;11(5).

128. Baving L, Laucht M, Schmidt MH. Atypical frontal brain activation in ADHD: preschool and elementary school boys and girls. J Am Acad Child Adolesc Psychiatry. 1999;38(11):1363-71.
